# Rhinoceros Feet Step Out of a Rule-of-Thumb: A Wildlife Imaging Pioneering Approach of Synchronized Computed Tomography-Digital Radiography

**DOI:** 10.1371/journal.pone.0100415

**Published:** 2014-06-25

**Authors:** Gabriela Galateanu, Robert Hermes, Joseph Saragusty, Frank Göritz, Romain Potier, Baptiste Mulot, Alexis Maillot, Pascal Etienne, Rui Bernardino, Teresa Fernandes, Jurgen Mews, Thomas Bernd Hildebrandt

**Affiliations:** 1 Department of Reproduction Management, Leibniz Institute for Zoo and Wildlife Research, Berlin, Germany; 2 ZooParc de Beauval, Saint-Aignan, France; 3 Parc zoologique d'Amnéville, Amnéville-les-Thermes, France; 4 Parc zoologique de La Barben (Pélissane), La Barben, France; 5 Hospital Veterinário, Jardim Zoológico de Lisboa, Lisbon, Portugal; 6 Clinical Application Research Center, Toshiba Medical Systems Europe, Zoetermeer, The Netherlands; INIA, Spain

## Abstract

Currently, radiography is the only imaging technique used to diagnose bone pathology in wild animals situated under “field conditions”. Nevertheless, while chronic foot disease in captive mega-herbivores is widely reported, foot radiographic imaging is confronted with scarcity of studies. Numerous hindrances lead to such limited numbers and it became very clear that the traditional perspective on bone imaging in domestic animals based on extensive studies and elaborated statistical evaluations cannot be extrapolated to their non-domestic relatives. For these reasons, the authors initiated a multi-modality imaging study and established a pioneering approach of synchronized computed tomography (CT) and digital radiography (DR), based on X-ray projections derived from three-dimensional CT reconstructed images. Whereas this approach can be applied in any clinical field, as a case of outstanding importance and great concern for zoological institutions, we selected foot bone pathologies in captive rhinoceroses to demonstrate the manifold applications of the method. Several advances were achieved, endowing the wildlife clinician with all-important tools: prototype DR exposure protocols and a *modus operandi* for foot positioning, advancing both traditional projections and, for the first-time, species-related radiographic views; assessment of radiographic diagnostic value for the whole foot and, in premiere, for each autopodial bone; together with additional insights into radiographic appearance of bone anatomy and pathology with a unique, simultaneous CT-DR correlation. Based on its main advantages in availing a wide range of keystone data in wildlife imaging from a limited number of examined subjects and combining advantages of CT as the golden standard method for bone diseases' diagnostic with DR's clinical feasibility under field conditions, synchronized CT-DR presents a new perspective on wildlife's health management. With this we hope to provide veterinary clinicians with concrete imaging techniques and substantial diagnostic tools, which facilitate straightforward attainment and interpretation of field radiography images taken worldwide.

## Introduction

Diagnostic imaging in domestic animals has a long-established pedestal on a plethora of published data supported by huge numbers (tens of thousands) of examined subjects. Not so is the situation for their wild counterparts. To illustrate this present and huge discrepancy, we purposely chose the most frequently applied imaging procedure in large animals, foot radiography, and compared between the most studied large mammals on land, the horse, as a representative for domestic animals, and the elephant, as a representative for wild animals. The only foot radiographic studies with indicated numbers of subjects found in *Elephantidae* (n = 4) included, in total, 15 elephants, with the largest number being 11 individuals per study [1], [2], [3], [4]. Nonetheless, an identical number of *Equidae* foot radiographic studies (n = 4), elected from 216 currently recorded publications, included 995 horses, with the largest number being 523 subjects per study [5], [6], [7], [8].

All-important hindrances lead to such scarce numbers of radiographic studies in wild animals, especially mega-herbivores. Among them can be mentioned: difficulty in access to free-ranging or captive wild animals [9], their untamed disposition implying serious risks in approaching them [10], [11], temporal constraints and survival risks imposed by prerequisite sedation and/or general anesthesia [12], [13], [14], tendency to disguise any sign of disease or clinical symptoms until late stages when they cannot be concealed any longer [15], [16], and difficulty of performing and interpreting radiographic examinations under “field conditions” [17]. These numbers decrease further in two additional circumstances. One condition is radiographic positioning intricacy due to massive body size of mega-vertebrates [18], [19]. The other situation is the intrinsic value of endangered wild animals, some of them being “the last of their kind”, as can be seen in rhinoceroses [20], [21], [22], [23], [24], [25], [26], [27], [28], [29]. Under these circumstances, any procedures that necessitate physical restraint, handling, transportation, sedation and/or general anesthesia will require a profound clinical justification, and thus are rarely performed. These challenges account for radiologic under-diagnosis of foot pathology in large-sized mammals [30].

Yet, chronic foot disease in captive herbivores is widely reported [10], [31], [32]. Remarkable evidence suggesting that foot osteopathology in hoofed mammals is more widespread, severe and diverse than previously thought [30], [33] should force us to rethink of radiographic diagnosis in captive mega-herbivores as routine examination to be incorporated into their health management. At any rate, apart from the elephant [2], [18], [19], [34], radiographic techniques, imaging protocols, and radiographic interpretation of foot bone anatomy and/or pathology in mega-vertebrates have not been established to date.

It became very clear that the traditional perspective on bone imaging in domestic animals based on extensive studies and elaborated statistical evaluations cannot be extrapolated to their non-domestic relatives. A new imaging strategy for assessment of different pathologies in wild animals became imperative and it is thus called for.

On this account, the authors initiated a comprehensive study, based on multi-modality imaging. We established a pioneering approach of synchronized computed tomography (CT) and digital radiography (DR), providing a new perspective on wildlife management. Whereas this approach can be applied in any clinical field, as a case in point, we selected one disease of outstanding importance: foot bone pathologies in wild animals. For this reason, synchronized CT-DR is demonstrated here using rhinoceros feet to show the manifold applications of the method. With this we hope to provide veterinary clinicians with concrete imaging techniques and substantial diagnostic tools which will facilitate straightforward implementation and interpretation of field radiographic images from rhinoceros feet taken worldwide. Without such advances, wildlife imaging will remain under the rule-of-thumb, now prevailing by necessity.

## Materials and Methods

### Ethics Statement

The four rhinoceroses (two Southern white and two Indian) included in our study were captive animals from the following zoological gardens: Parc zoologique d'Amnéville, France; Parc zoologique de La Barben (Pélissane), France; ZooParc de Beauval, France; and Jardim Zoológico de Lisboa, Portugal. Southern white rhinoceros is listed under the IUCN the Red List of Endangered Species as Near Threatened and the Indian rhinoceros is listed as Vulnerable. These animals either died (rhinoceros 4: metastasized adenocarcinoma) or were euthanized due to chronic, non-resolvable health issues and subsequent animal welfare reasons, following internal decision-making process in the respective zoos (rhinoceros 1: foot epidermoid carcinoma with 3^rd^ grade lameness; rhinoceros 2: generalized chronic ulcerative dermatitis; rhinoceros 3: chronic pododermatitis and recumbency without movement). The euthanasia procedures were performed in conformity with the international guidelines for euthanasia in non-domestic species, specifically for mega-vertebrates [35]. In accordance with these guidelines, animals were first immobilized with etorphine hydrochloride to achieve full recumbent anesthesia. Euthanasia was then achieved by intravenous administration of a barbiturate.

No animal work was involved at any stage in the process and all samples (distal feet) were collected after the unrelated death of the animals. The zoos were approached upon our learning of the animals' death and gave their permission to use the feet for this study, in the context of mandatory *post mortem* examination and disease diagnosis. This *post mortem* diagnostic study was in accordance with the guidelines of the Internal Committee of Ethics and Animal Welfare of the Leibniz Institute for Zoo and Wildlife Research as stipulated under approval number 2006-01-02.

### Rhinoceroses

Ten distal limbs (five front and five hind legs) obtained *post mortem* from four captive rhinoceroses, were used for this study (Table 1). The rhinoceroses were of two species: Southern white rhinoceros (*Ceratotherium simum simum*) and greater one-horned, or Indian, rhinoceros (*Rhinoceros unicornis*). Distal limb encompassed the autopodium (and its related soft-tissue structures) represented by the hand (manus) or foot (pes), being composed of podial elements (carpus/tarsus), metapodials (metacarpus/metatarsus) and phalanges [36].

**Table 1:** Rhinoceroses.

Rhinoceroses	Species	Gender	Age (Years)	Feet
**Rhinoceros 1**	*Ceratotherium simum simum*	Male	38	HL
**Rhinoceros 2**	*Ceratotherium simum simum*	Male	38	FR, FL, HL
**Rhinoceros 3**	*Rhinoceros unicornis*	Female	24	FR, FL, HR, HL
**Rhinoceros 4**	*Rhinoceros unicornis*	Female	34	FL, HR

*Ceratotherium simum simum*- Southern white rhinoceros, *Rhinoceros unicornis*- Indian rhinoceros, FR - front right, FL - front left, HR - hind right, HL - hind left autopodium.

Rhinoceros 1 (Southern white rhinoceros) presented a medial, large tumefaction on its hind left foot, diagnosed histologically as epidermoid carcinoma. Rhinoceros 3 (Indian rhinoceros) suffered from chronic pododermatitis in all four limbs for many years. Rhinoceroses 2 (Southern white rhinoceros) and 4 (Indian rhinoceros) had no reported foot disease. Rhinoceroses 1 and 3 were euthanized due to foot related disorders and rhinoceros 2 was euthanized and rhinoceros 4 died due to other, unrelated, pathologies.

The legs of the two Southern white rhinoceroses were sectioned above the carpal and tarsal joints (included). Except for the hind foot of rhinoceros 4, Indian rhinoceroses' legs were sectioned at the level of carpal and, respectively, tarsal joints (partially included). Therefore, the total number of bones included in this study was 257 instead of 278.

### Computed tomographic data acquisition and imaging

Computed tomographic data was acquired from all ten distal limbs using a high-resolution, 128-slice scanner (Aquilion CX, Toshiba Medical Systems Cooperation, Tochigi, Japan). Settings for the CT helical scan protocol were: 120 kV, 100–300 mA, 0.6 s rotation time, helical pitch HP 41.0 and 0.5 mm acquisition slice thickness. Reconstruction protocols included two soft tissue reconstructions (body-standard and body-sharp) and a high-resolution reconstruction algorithm for bones. The reconstruction slice thickness/slice interval of both was set to 1/0.8 mm and 0.5/0.25 mm.

Vitrea workstation with ViTREA 2 version 4.0 medical diagnostic software (Vital Images Inc., Minnetonka, MN, USA) provided the tools for two-dimensional (2D) and three-dimensional (3D) processing and analysis of the CT images. Among these tools, volume-rendering software, simultaneous imaging of specific anatomical and pathological structures of interest using a combination of 2D orthogonal Multi-Planar Reconstructions (MPR) and 3D images; a virtual cutting function in combination with 2D and 3D segmentation allowed us to focus on the region of interest. A wide variety of clinical viewing protocols and fine adjustment of visualization parameters, e.g. adjustments of threshold and transparency settings enhanced the diagnostic quality of the images. Oblique and curved MPRs were required in order to delineate several lesions with a complex 3D architecture.

### Synchronized computed tomography and digital radiography

Fully rendered volumetric (3D) CT images were acquired from all ten feet. Based on a predefined sectional plane of the object, synchronized X-ray projections were calculated and generated by applying specialized software tools on the image console.

**Figure 1:**
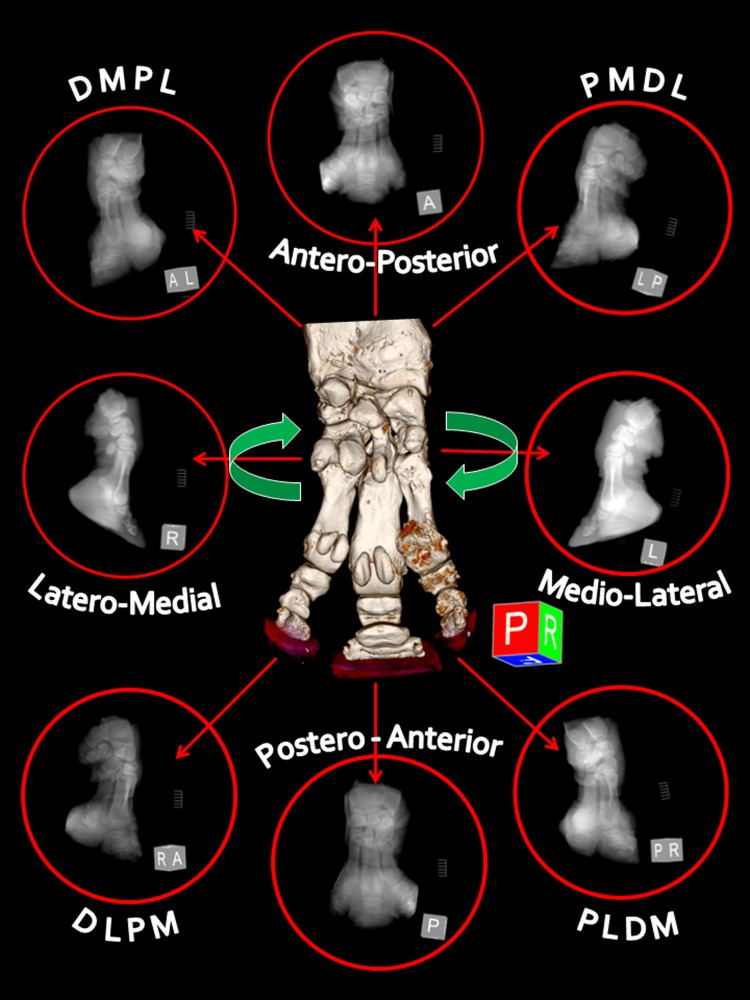
Principle of method in synchronized computed tomography (CT)-digital radiography (DR). Digital radiographic images are calculated and generated from fully rendered, tri-dimensional (3D) CT images. Standard orthogonal (n = 4) and oblique (n = 4) radiographic views (45° apart) are shown here using as example the left front distal limb of Southern white rhinoceros 2. The abbreviations used represent the oblique projections characterized by the point-of-entry to point-of-exit direction of the primary X-ray beam: DMPL [dorsomedial-palmarolateral], PMDL [palmaromedial-dorsolateral], PLDM [palmarolateral-dorsomedial], and DLPM [dorsolateral-palmaromedial].

For each foot, eight 3D CT images (45° apart) equivalent to eight standard radiographic views were generated. In order to simulate DR views, the acquired CT datasets were used to generate renderings from 8 different viewing directions for each foot. Thus, each 3D CT image was transformed into a synchronized digital radiographic image (Synch DR), in total eighty Synch DR images for all ten feet (Fig.1).

The standardized nomenclature for radiographic projections in veterinary medicine was used [37], [38]. The projections performed, indicating point-of-entry to point-of-exit direction of the primary X-ray beam, were four orthogonal projections: dorso-palmar (plantar) [DPa(l)], palmaro (plantaro)-dorsal [Pa(l)D], medio-lateral [ML], latero-medial [LM], and four oblique projections: dorsomedial-palmaro (plantaro) lateral [DM-Pa(l)LO], dorsolateral-palmaro (plantaro) medial [DL-Pa(l)MO], palmaro (plantaro) medial-dorsolateral [Pa(l)M-DLO] and palmaro (plantaro) lateral-dorsomedial [Pa(l)L-DMO]. For simplification reasons, “P” was used for either Pa (palmar) or Pl (plantar), when it was not relevant if it is front or hind foot.

Windowing and leveling of each Synch DR were further adjusted in order to obtain the best radiographic quality in terms of resolution, contrast and noise. These Synch DR images were designated as gold standard images and were used as reference in establishing the most accurate positioning and appropriate exposure parameters for direct digital radiography (DR).

### Direct digital radiography

Traditional DR was conducted on all nine distal limbs from rhinoceroses 2, 3 and 4, using a mobile x-ray unit (Mobi X-Ray, SEDECAL, Madrid, Spain) and Canon CXDI-1 image plate (Canon CXDI-1 System Digital Radiography; CANON Europe N.V. Medical Products Division, Amstelveen, The Netherlands).

Different radiographic projections were achieved by maintaining the X-ray generator and image plate in the same position, while rotating the foot. The foot was positioned parallel to and in the nearest proximity of the image plate.

An optimal exposure chart was established showing the relationship between different radiographic views and the exposure values: miliampere (mA), kilovolt peak (kVp), time (s), at a constant source-to-film or focus-to-film distance (FFD) of 100 cm.

Different anatomical landmarks and radiographic planes were investigated to nominate reference indicators for foot positioning and the outcome is presented in the “Results” section.

For each foot, eight radiographic views were performed in accordance with the gold standard Synch DR images established before. Seventy-two DR were thus assessed for depiction of bone anatomy and pathology.

### Digital radiographic evaluation

Two criteria were investigated in each radiographic view:

a) Number of bones that were discernible at a diagnostic value (presented as percentage from the total number of foot's bones);

b) Perceptible radiographic details of each bone estimated with a 5-point radiographic rating scale. The following values were used, from 1 to 5: 1 =  deficient (many bones superimposed and no detail), 2 =  inadequate (three or more bones superimposed, poor detail), 3 =  satisfactory (two bones superimposed, but relatively good detail), 4 = good (minimal or partial superimposition, good detail), 5 =  excellent (minimal superimposition, very good detail).

Diagnostic value of every radiographic view was assessed solely for each bone and, by summation, entirely for the whole foot.

### Conventional analogic radiography

Sixteen plain or analog radiographs (AR) of all feet, including four radiographic views per foot were performed and manually developed in rhinoceros 3, using a HF 300 X-ray unit (GmbH Gierth), X-Omat radiographic cassette, Kodak Lanex Medium Screen and Kodak T-Mat L/RA radiographic films. The exposure parameters were: 40 mA, 76 kVp, 0.06 s for all projections of hind feet and medio-lateral projection of front feet; 40 mA, 74 kVp, 0.06 s for the rest of front feet's projections, all at a constant FFD of 80 cm.

### Statistical analysis

Statistical analysis was performed using PASW Statistics 18 (formerly SPSS, IBM Inc., Chicago, IL). The Chi-square goodness-of-fit exact test was used to test whether the observed proportions for categorical variables differ from the hypothesized equal distribution.

Rhinoceros 1 suffered from epidermoid carcinoma on the only limb available from this animal. As this tumor may have been the cause for at least some of the osteopathologies found in this foot and thus may have biased the statistical analysis, we have also analyzed our data after excluding this animal. Results indicate no biasing effect of rhinoceros 1 as none of the comparisons changed in a way that alter our findings (data not shown). Results are therefore shown for all four rhinoceroses combined.

A *P*-value <0.05 was considered statistically significant for all statistical tests.

## Results

### Reference radiographic techniques

**Figure 2:**
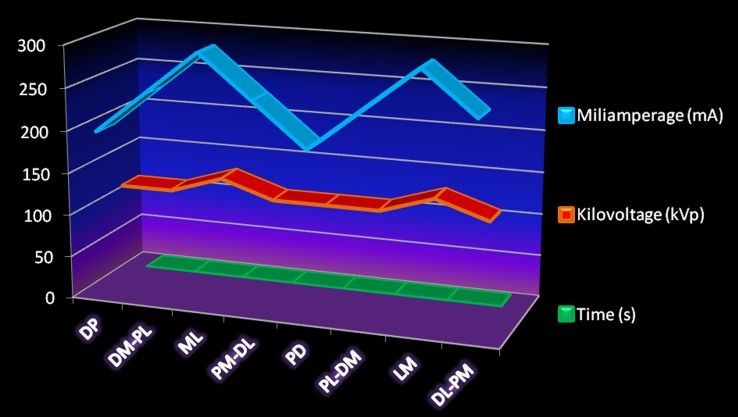
Radiographic exposure chart for front and hind feet in both Southern white and Indian rhinoceroses. On the horizontal axis are the eight radiographic views and the vertical axis shows the exposure values of: milliampere (mA), kilovolt peak (kVp) and time (s) for each projection at a constant source-to-film, or focus-to-film, distance (FFD) of 100 cm. Standard radiographic views were: DP [dorso-palmar (plantar)], DM-PL [dorsomedial-palmaro (plantaro) lateral], ML [medio-lateral], PM-DL [palmaro (plantaro) medial-dorsolateral], PD [palmaro (plantaro)-dorsal], PL-DM [palmaro (plantaro) lateral-dorsomedial]; LM [latero-medial], DL-PM [dorsolateral-palmaro (plantaro) medial].

Exposure parameters (mAs, kVp) were similar for both Southern white and Indian rhinoceroses, with no differences between front and hind feet, as can be seen in the proposed technique chart (Fig. 2). The highest exposure factors were required for the ML/LM views, whereas the lowest exposure was entailed for PD/DP views. Oblique views called for intermediary exposure parameters. An alternative exposure chart is also proposed, applying a longer exposure time. The main advantages of this alternative protocol are lower kVp and higher mA, leading to an improved bone imaging (Fig. S1). Additionally, in this variant, all oblique and DP/PD orthogonal projections could be performed with identical exposure.

The most reliable anatomical landmark found in both Indian and Southern white rhinoceros was the toenail of the third (central) digit. Radiographic projections obtained at angles of 45°, or multiples of it, from the reference dorsal mid-line passing through the central toenail, were as follows: DP —^45°^— DM-PL —^45°^— ML — ^45°^— PM-DL — ^45°^— PD — ^45°^— PL-DM — ^45°^— LM —^45°^— DL-PM — ^45°^— DP (Fig. 1).

**Figure 3:**
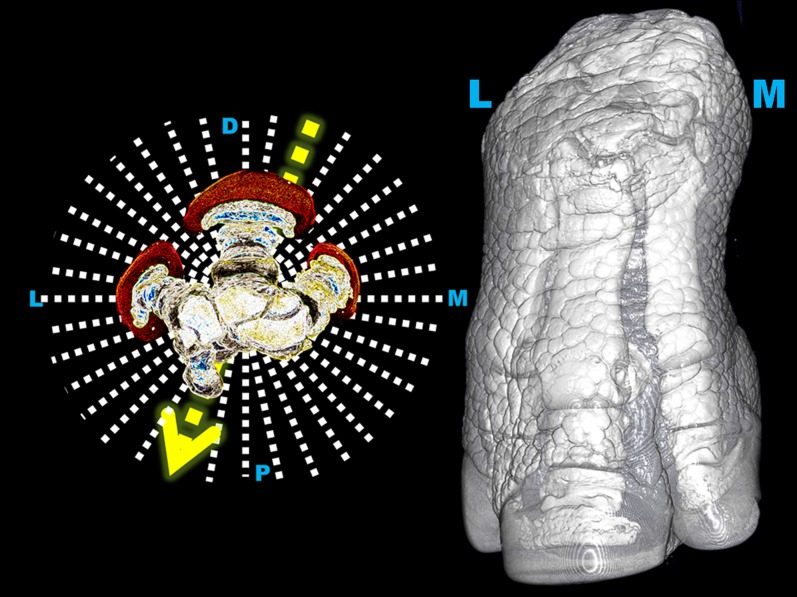
Rhinoceroses' species-related radiographic views. Dorsomedial-palmarolateral (DMPL) 20° oblique view performed at a projection angle of 20° from the dorsal mid-plane (arrow) allows a better visualization of all digits than the traditional DMPL 45° oblique view. Positioning technique is demonstrated on tri-dimensional computed tomographic (3D CT) image of Indian rhinoceros 3 right front foot (right side image) and schematically represented using a cross-sectional CT image (left side image). Semi-transparent 3D CT imaging protocol was employed to show both foot's exterior aspect and the underlying bony structures.

Specifically for digits, Synch DR revealed that, in order to achieve minimal bone super-imposition, projection angles must have different values in rhinoceroses than the traditional projections known from domestic radiography. Starting with DP view, these were the optimal angles for the front foot: DPa —^20°^— DM-PaL —^60°^— ML — ^70°^— PaM-DL — ^30°^— PaD — ^20°^— PaL-DM — ^70°^— LM —^60°^— DL-PaM — ^30°^— DPa (Fig. 3 and Fig. S2-8). Starting with DP view, these were the optimal angles for the hind foot: DPl —^20°^— DM-PlL —^70°^— ML — ^70°^— PlM-DL — ^20°^— PlD — ^20°^— PlL-DM — ^70°^— LM —^70°^— DL-PlM — ^20°^— DPl. By comparison, rhinoceroses' specific projection angles revealed higher numbers of detected digits' osteopathologies than the traditional angles, in all feet.

### Radiographic diagnostic value

**Figure 4:**
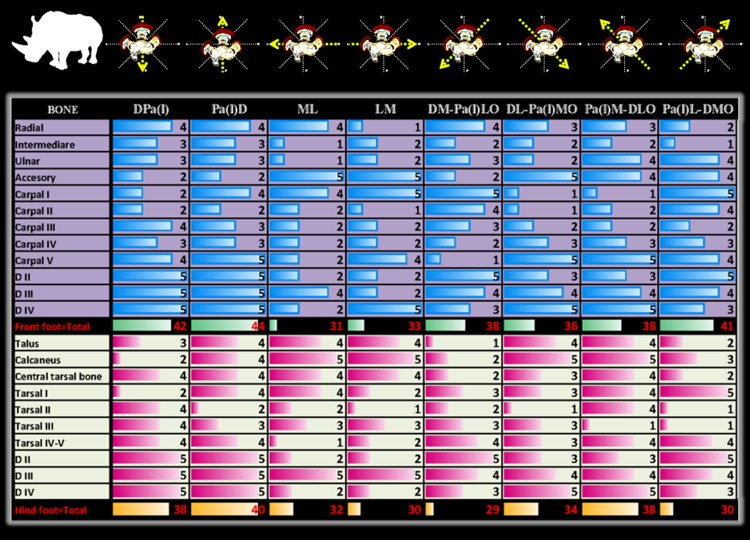
One-criterion diagnostic value of traditional (45° and multiples of 45° projection angles) radiographic views. Radiographic diagnostic value per view was calculated by summation of the perceptible radiographic detail assessed with a 5-point rating scale for each autopodial bone of front and, respectively, hind feet in Southern white rhinoceros. Standard radiographic views are schematically represented on the top row. Abbreviations: digits II, III, IV [D II, D III, D IV]; views: dorso-palmar (plantar) [DPa(l)], palmaro (plantaro)-dorsal [Pa(l)D], medio-lateral [ML], latero-medial [LM], and four oblique projections: dorsomedial-palmaro (plantaro) lateral [DM-Pa(l)LO], dorsolateral-palmaro (plantaro) medial [DL-Pa(l)MO], palmaro (plantaro) medial-dorsolateral [Pa(l)M-DLO] and palmaro (plantaro) lateral-dorsomedial [Pa(l)L-DMO].

**Figure 5:**
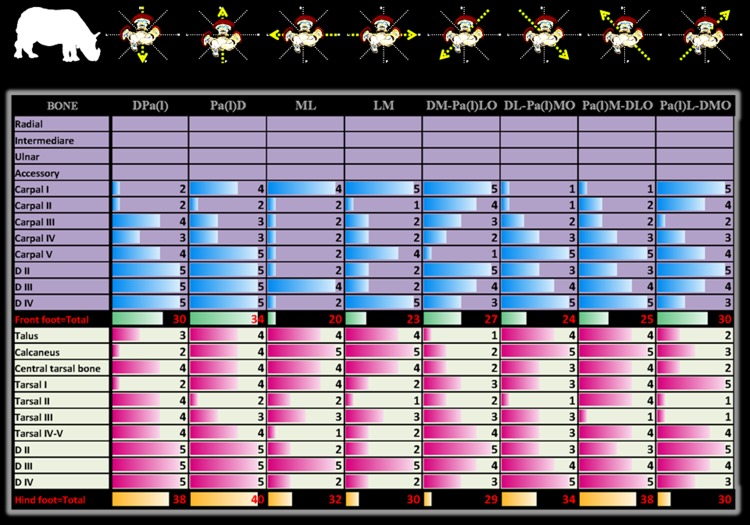
One-criterion diagnostic value of traditional (45° and multiples of 45° projection angles) radiographic views. Radiographic diagnostic value per view was calculated by summation of the perceptible radiographic detail assessed with a 5-point rating scale for each autopodial bone of front and, respectively, hind feet in Indian rhinoceroses. Standard radiographic views are schematically represented on the top row. Abbreviations: digits II, III, IV [D II, D III, D IV]; views: dorso-palmar (plantar) [DPa(l)], palmaro (plantaro)-dorsal [Pa(l)D], medio-lateral [ML], latero-medial [LM], and four oblique projections: dorsomedial-palmaro (plantaro) lateral [DM-Pa(l)LO], dorsolateral-palmaro (plantaro) medial [DL-Pa(l)MO], palmaro (plantaro) medial-dorsolateral [Pa(l)M-DLO] and palmaro (plantaro) lateral-dorsomedial [Pa(l)L-DMO].

**Figure 6:**
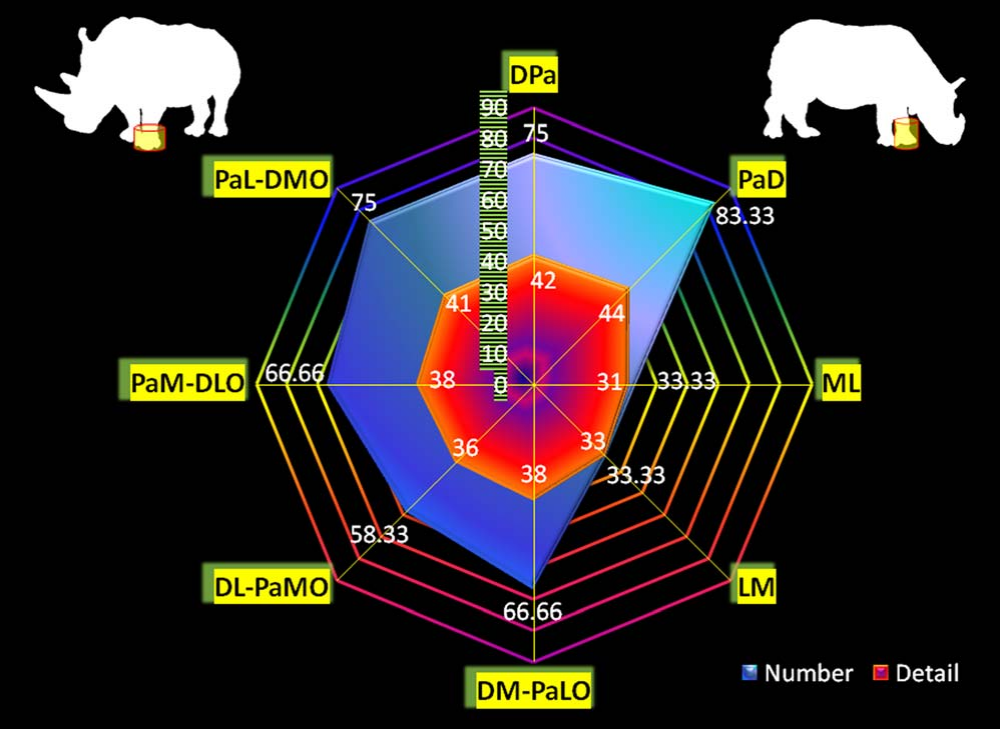
Two-criteria diagnostic value of traditional (45° and multiples of 45° projection angles) radiographic views. Whole foot radiographic diagnostic value per view was calculated based on: a) number of bones that could be discerned at a diagnostic value (“Number”, presented as percentage from the total number of foot's bones); b) perceptible radiographic details of each bone estimated with a 5-point radiographic rating scale and summated for all foot's bones (“Detail”). The results are shown for front feet in Southern white and Indian rhinoceroses. The abbreviations used are: dorso-palmar (plantar) [DPa(l)], palmaro (plantaro)-dorsal [Pa(l)D], medio-lateral [ML], latero-medial [LM], and four oblique projections: dorsomedial-palmaro (plantaro) lateral [DM-Pa(l)LO], dorsolateral-palmaro (plantaro) medial [DL-Pa(l)MO], palmaro (plantaro) medial-dorsolateral [Pa(l)M-DLO] and palmaro (plantaro) lateral-dorsomedial [Pa(l)L-DMO].

**Figure 7:**
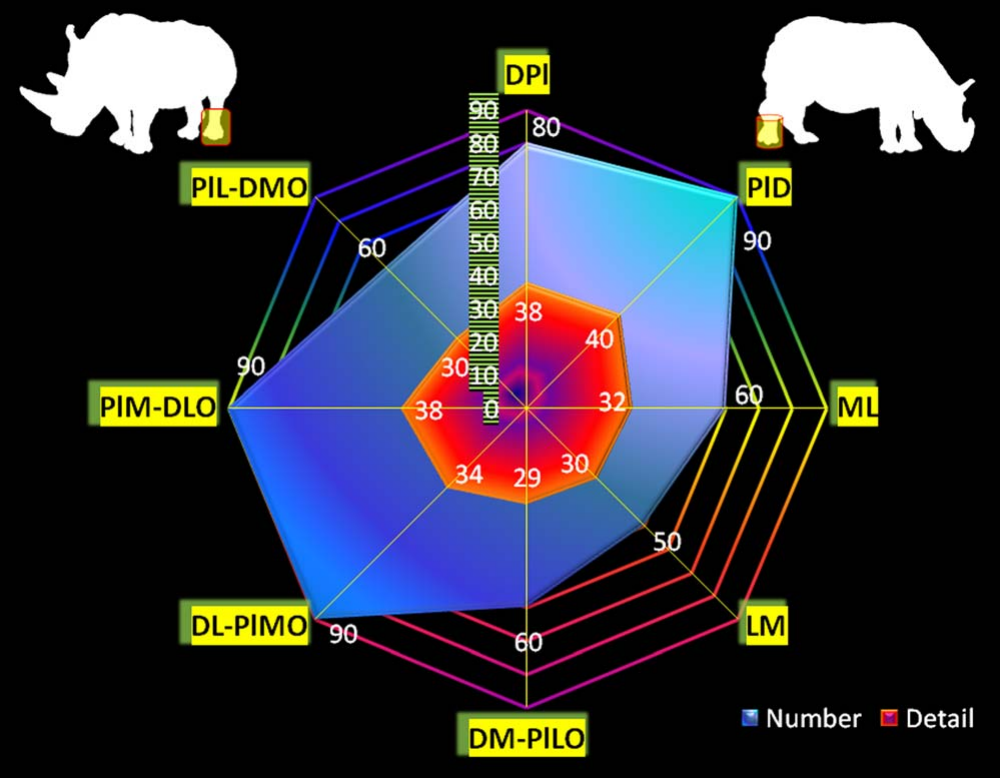
Two-criteria diagnostic value of traditional (45° and multiples of 45° projection angles) radiographic views. Whole foot radiographic diagnostic value per view was calculated based on: a) number of bones that could be discerned at a diagnostic value (“Number”, presented as percentage from the total number of foot's bones); b) perceptible radiographic details of each bone estimated with a 5-point radiographic rating scale and summated for all foot's bones (“Detail”). The results are shown for hind feet in Southern white and Indian rhinoceroses. The abbreviations used are: dorso-palmar (plantar) [DPa(l)], palmaro (plantaro)-dorsal [Pa(l)D], medio-lateral [ML], latero-medial [LM], and four oblique projections: dorsomedial-palmaro (plantaro) lateral [DM-Pa(l)LO], dorsolateral-palmaro (plantaro) medial [DL-Pa(l)MO], palmaro (plantaro) medial-dorsolateral [Pa(l)M-DLO] and palmaro (plantaro) lateral-dorsomedial [Pa(l)L-DMO].

Except for the first carpal row in Indian rhinoceroses (not included), radiographic detail value of each bone per radiographic view was identical for Southern white and Indian rhinoceroses (Fig. 4, 5). Likewise, the number (percentage) and, respectively, radiographic detail of autopodial bones as a unit per view were identical for Southern white and Indian rhinoceroses (Fig.6, 7). Additionally, in Southern white and Indian rhinoceroses, the radiographic projection with the highest diagnostic value for both front and hind feet was the PD view (Fig.6, 7).

Comparison of the diagnostic value (depicted bones' number and detail) of different radiographic views revealed dissimilar patterns between front and hind feet (Fig.6, 7). The most valuable views in terms of number of depicted bones, represented as percentage from the total number of autopodial bones, were PaD (83.3%), DPa (75%), PaL-DMO (75%), for the front foot and PlD (90%), DL-PlMO (90%), PlM-DLO (90%), for the hind foot. The most valuable views in terms of radiographic detail, represented as total units in 5-point radiographic scale, were PaD (44), DPa (42), PaL-DMO (41), for the front foot and PlD (40), DPl (38), PlM-DLO (38), for the hind foot.

Distinctly, there was not always a direct relationship between the two parameters (number and discernible detail) for a specific foot's radiographic view. As a case in point, for the hind foot, PlD, DL-PlMO, and PlM-DLO views provide information about the same number of bones (90% discernible bones), but the radiographic detail is higher on PlD view (40 points in the rating scale), followed by PlM-DLO (38 points in the rating scale) and DL-PlMO (34 points in the rating scale).

Additionally, no relationship was found between the diagnostic values for the whole autopodium and for any specific bone. For example, first carpal bone was very well visualized (5 in the rating scale) in DM-PaLO view, but indiscernible (1 in the rating scale) in PaM-DLO view, though both projections had the same overall diagnostic value (66.66% discernible bones and 38 points in the rating scale).

### Bone anatomy and pathology in computed tomographic imaging

**Figure 8:**
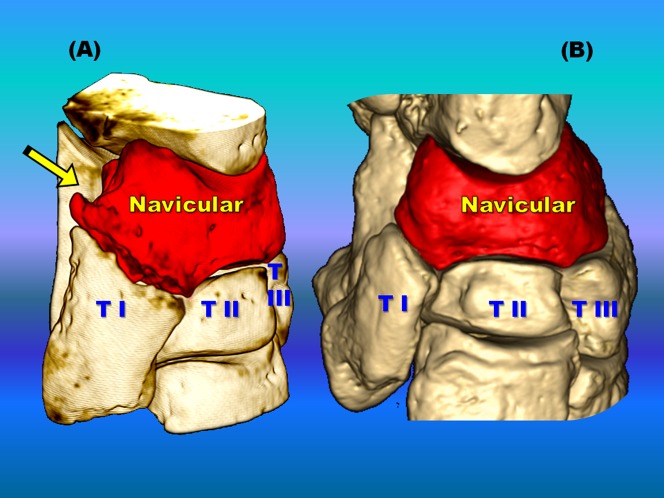
Left navicular (central tarsal bone, CTB) comparative imaging in two Southern white rhinoceroses. Tri-dimensional computed tomographic (3D CT) images of CTB allowed comparison of multiple bone pathologies (A) in rhinoceros 1 with normal anatomical aspect (B) in rhinoceros 2. Encountered osteopathologies are: cortical osteogenesis represented by massive, unstructured new bone production and remodelling, with a beak-like formation oriented plantaro-medially (arrow). Additionally (A), the articular surface between CTB and first tarsal bone (TI) is highly irregular, characterized by decreased joint space width and articular bone proliferation that bridges the contiguous bones (ankylosis). The second (TII) and the third (TIII) tarsal bones are within normal limits on both rhinoceroses (A, B).

Computed tomographic images depicted both bone anatomy and pathology (Fig. 8). A total of 257 autopodial bones were investigated in this study. Among them, 69 bones (26.8%) at 117 sites in all Indian and Southern white rhinoceroses presented pathological changes. These comprised of a large spectrum of lesions including cortical sclerosis (Fig. S9), proliferative new bone formation and bone remodeling (Fig. S10) with loss of normal shape (33/117; 28.2%), intra- and periarticular mineralized bodies or bony fragments (27/117; 23.1%; Fig. S11), fractures (19/117; 16.2%; Fig. S12), periosteal proliferation (continuous and interrupted; 19/117; 16.2%; Fig. S13), osteolysis and bone rarefaction (13/117; 11.1%). Enlargement of the linear radiolucent areas along the distal border of the distal phalanx termed “vascular channels”, and changes in the trabecular pattern were also found. Bone cystic formation (n = 4) and ankylosis (n = 2) were the rarest osteopathologies. Concomitant presence of several lesions was similar in appearance to end stage degenerative joint disease (DJD), osteoarthrosis and/or osteoarthritis. Of the 117 sites with bone pathologies, significantly more were situated in the front limbs than in the hind limbs (n = 72 vs. n = 45, respectively; Chi-square  = 6.231, P = 0.016). Comparison between the medial and lateral digits revealed a higher prevalence of osteopathologies on the medial digit in the hind (n = 18 vs. n = 7; Chi-square  = 4.840, P = 0.043) but not in the front (n = 27 vs. n = 20; Chi-square  = 1.043, P  = 0.382) limbs. The third or middle digit was less affected than the medial digit in the hind limbs (n = 18 vs. n = 5; Chi-square  = 7.348, P = 0.011) as well as in the front limbs (n = 27 vs. n = 11; Chi-square  = 6.737, P = 0.014). When prevalence of osteopathologies per digit was compared for both front and hind limbs combined, there were more osteopathologies in the medial digit (n = 45) when compared to the lateral digit (n = 27; Chi-square  = 4.500, P = 0.044) or the middle digit (n = 16; Chi-square  = 13.787, P = 0.00026). While the medial digit presented more osteopathologies when compared to the lateral digit, this was not the case when the middle, or third, digit was compared to the lateral one. No difference was found in either front or hind limbs or if both front and hind feet were combined when prevalence of osteopathologies was compared between the middle and lateral digits. The only difference found between the medial and lateral digits when osteopathologies' prevalence was compared was in the occurrence of periosteal reaction (n = 13 vs. n = 3, respectively; Chi-square  = 6.250, P = 0.021). There were also more periosteal reaction (n = 13 vs. n = 1; Chi-square  = 10.286, P = 0.00183) and bone remodelling (n = 15 vs. n = 1; Chi-square  = 12.250, P = 0.00052) in the medial digit when compared to the middle digit. The digits (including metapodial, phalangeal and sesamoidal bones) were by far the most prevalent site for osteopathologies, presenting more osteopathologies than in the podial elements (carpus and tarsus) combined (n = 88 vs. n = 29; Chi-square  = 29.752, P<0.00001). Of the digital elements, the phalanges constituted 77.2% of the lesions, metapodials 15.9% of the lesions, and proximal sesamoids 6.8% of the lesions. Within the digits, the highest prevalence of osteopathologies (54.4% of the lesions) was in the third phalanx (n = 37, with 19 lesions in the hind legs and 18 in the front legs), more than the second phalanx (n = 17; Chi-square  = 7.407, P = 0.0091) or the first phalanx (n = 14; Chi-square  = 10.373, P = 0.00177). There was no difference in osteopathologies prevalence between the first and second phalanges. The carpal and tarsal bones presented a wide variety of pathologies such as fractures, focal osteolysis, enthesiophytosis, osteophytosis, cortical osteogenesis, bone remodeling, and ankylosis.

### Bone anatomy and pathology in digital radiographic imaging

**Figure 9:**
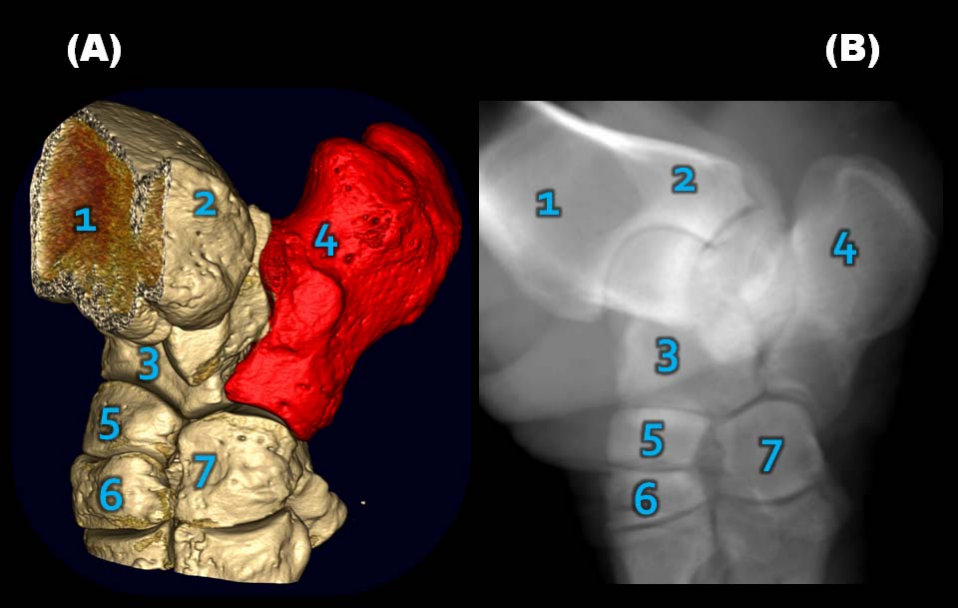
Tarsal normal anatomy depicted in Southern white rhinoceros 2 left hind foot by (A) computed tomography (CT) and (B) synchronized digital radiography (Synch DR). This projection (B) allows the best visualization of calcaneus (highlighted on CT image A) with minimal superimposition of other bony elements. The abbreviations used are: 1-tibia, 2- fibula, 3- talus, 4- calcaneus, 5- navicular, 6-tarsal III, and 7- tarsal IV bones.

**Figure 10:**
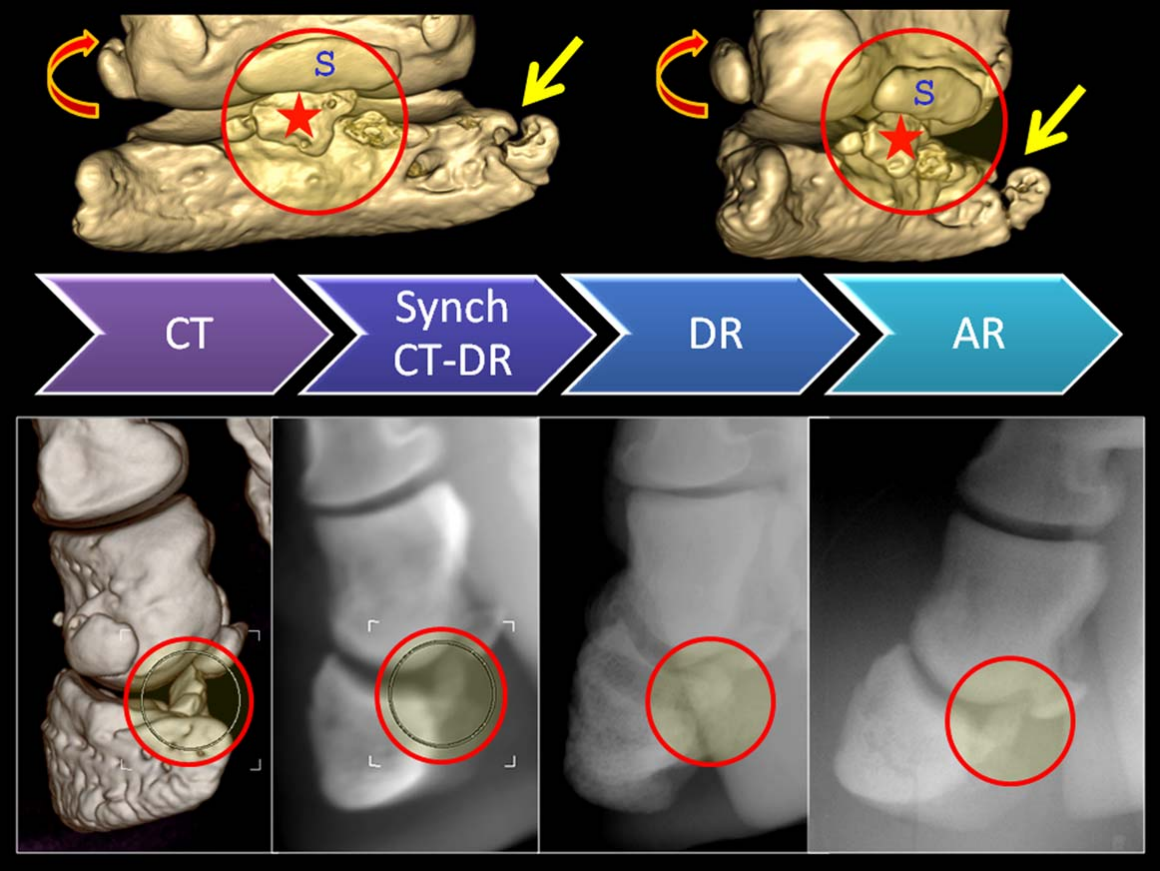
Multi-modality imaging diagnosis of bone pathology in Indian rhinoceros 1. Fractured bony fragments (circle and star) of the distal phalanx of the left front central digit are imaged ventral to the central small sesamoid (S) by means of: analog radiography (AR), digital radiography (DR), computed tomography (CT), and synchronized CT-DR (Synch CT-DR). Uppermost CT images reveal additional osteopathologies: the second phalanx - dorso-lateral fracture with a displaced fragment (curved arrow); the third phalanx - complete fracture of the medial palmar process (*processus palmaris medialis*, straight arrow).

Synchronized CT-DR depicted radiographic aspect of both normal anatomy (Fig. 9) and bone pathology (Fig. 10; Figs. S9-S13). Digital and conventional radiographic images gave clear information on numerous bone lesions as: specific fractures, ankylosis, osteolysis, extensive new bone proliferation, bone fragments or mineralized bodies, severe periosteal reaction, bone remodeling etc. Nevertheless, other lesions detected in CT images could not be depicted by digital or conventional radiographs (osseous fissure lines, small or subchondral bone fractures, fractures with a complicated 3D architecture, mild periosteal reaction, minor bone remodeling, and cortical sclerosis).

## Discussion

### Need for a novel approach in large wild animals' imaging

Computed tomography, as the golden standard for bone imaging [39], [40], [41], can be performed in very large animals merely on excerpts and only *post mortem* [19], [42], [43], [44]. This *ex situ* examination encounters several challenges related to harvesting, storage and transportation procedures. The only *ante mortem*, *in situ* imaging technique available to date for veterinary clinicians working under field conditions is radiography. Due to difficulties in approaching non-domestic animals, and especially mega-vertebrates, many diagnostic procedures are simply not done, overlooked, or performed too late.

Chronic foot disease, a devastating disorder generally thought to be confined to soft tissues, is widely reported [32], [45], [46] and a subject of concern for many zoological gardens owing to its severe impact on animal's general health [47], [48], [49]. Bearing in mind that the future for some species might be only in captivity, the importance of eradicating chronic foot disease never became as important as in captive wild animals. Hitherto, clinicians lacked the diagnostic imaging tools, namely radiographic techniques and protocols, as well as reference documentation regarding radiographic interpretation of both normal anatomy and pathology. In our opinion, the assumption as if chronic foot disease is due to soft tissue issues and the scarcity of data on foot bone pathology [50], [51], [52], [53] are due to lack of radiographic assessment. Therefore, recognizing the importance of looking at this area of science anew, we confronted it from a different, non-invasive perspective: imaging diagnosis. To this end, a pioneering approach of synchronized computed tomography and digital radiography was instituted [54], [55], [56]. Reported technical impediments (scarce number of animals, positioning intricacy, etc) have been met with success and the knowledge achieved can be used as a valuable groundwork for future radiographic studies.

### Tools offered by synchronized computed tomography and digital radiography

The main advantage of Synch CT- DR is its capability to provide a wide range of keystone data in wildlife imaging from a limited number of examined subjects. Additionally, it combines the advantages of CT as the golden standard method for bone diseases' diagnostic with DR's clinical feasibility under field conditions. Several advances were achieved from this pioneering approach, providing the wildlife clinician with all-important tools:

- Prototype digital radiographic exposure protocols and a *modus operandi* for foot positioning, advancing both traditional projections and first-time, species-related radiographic views;
- Radiographic diagnostic value for the whole foot and, in premiere, for each autopodial bone;
- Additional insights into radiographic appearance of bone anatomy and pathology with a unique CT-DR correlation.

### Reference radiographic techniques

It is indisputable that dissimilar radiographic techniques will lead to reporting inconsistency, and any comparison of the already scarce data will therefore be impossible. Conversely, use of a consistent technique will facilitate case consultations and comparative, inter-institutional imaging studies.

This study was designed to identify the relevant radiographic views and proper exposure parameters for accurate depiction of normal anatomy and pathological changes in the rhinoceros foot.

Several aspects must be taken into consideration, as follows. Hoof's preparation is of the utmost importance in eliminating several artifacts and producing radiographs of diagnostic quality [57]. Considering the uniqueness of each X-ray generator and detector combination, clinicians need to develop their own techniques for obtaining good radiographic quality [58]. Presented exposure charts are offered as reference. Adjustment of these techniques should be made, taking into consideration the animal's weight and size, foot's condition and the pathology involved (bone versus soft tissue). Bearing in mind the large size of the rhinoceros, positioning for various studies should be performed by rotating a portable radiographic unit (X-ray beam) and the image plate at required angles corresponding to each projection. Beam-plate angles of 90° were used, but other angles should be investigated because they may reveal more appropriate projections, as it was found in horses [59], [60] and, very recently, in elephants [61]. Projection angles of 45° between different radiographic views will give detailed foot evaluation. Nevertheless, this study showed that species dependent anatomical variations must be taken into account. Due to rhinoceros' special foot anatomy, traditional positioning using, as accurate landmarks, specific anatomical structures could not be applied. Distinctive features responsible for difficulty in visualization and palpation of any anatomical landmark include: considerable skin thickness, distal leg's bulk and cylindrical shape, massive central foot pad, digits' largest part (metapodials and first phalanges) included into compact soft tissue mass and, especially, the asymmetric arrangement of the medial and lateral digits in the front foot. Therefore, foot positioning was performed taking as reference line the dorsal aspect of the mid-sagittal plane (perpendicular on and passing through the middle of the third digit). The central (third digit) toenail was used as anatomical landmark. New radiographic views were established for a better visualization of the rhinoceros' digits while avoiding or minimizing superimposition of the large sesamoids on the metapodial and phalangeal elements. Distinctive anatomy of rhinoceros' front foot, with medial metacarpus (Mc II) being rotated inwardly along its vertical axis and positioned more palmary in comparison with the central metacarpal bone (Mc III), accounted for the differences encountered on projection angles between front and hind legs.

### Radiographic diagnostic value

Unlike articulations between long bones, carpal and tarsal joints are considerably polyostotic, with complex 3D general architecture and complicated, multi-facet bone geometry. For these reasons, an accurate evaluation of these joints requires multiple radiographic views. Most commonly, eight radiographs per foot, with four orthogonal and four oblique projections are insufficient for reliable podial assessment, frequently necessitating additional views: hyperflexion, hyperextension, adduction, abduction, sky-views etc. Unlike their domestic relatives, these all-inclusive standard procedures are difficult to perform in wild animals due to the need for sedation or anesthesia, and temporal and positioning intricacies. Thus, the clinician will benefit from an exhaustive knowledge of the diagnostic potential of each radiographic view, making possible the establishment of high-priority views to start with. The present study endows with data on diagnostic value of each radiographic projection, in general, and for each autopodial bone in particular (excepting first carpal row in Indian rhinoceroses, not included). A comparative study of segregated first row carpal bones revealed minimal morphologic differences between Southern white and Indian rhinoceroses. Therefore, the radiographic diagnostic values were extrapolated from Southern white to Indian rhinoceroses for radial, intermediary, ulnar and accessory carpal bones.

### Multi-modality comparative imaging study

The results of our study indicate that bone lesions were present in both Indian and Southern white rhinoceros species. Reported bone pathology comprises a wide spectrum of lesions affecting a large number of autopodial bones. It was encountered in rhinoceroses with soft tissue tumour or with known chronic foot disease (rhinoceroses 1, 3) and, most surprisingly, in a Southern white rhinoceros (rhinoceros 2) and an Indian rhinoceros (rhinoceros 4) that showed no discernible clinical signs of foot afflictions. Possible origins, prevalence and distribution of foot bone pathology were discussed previously [30].

This study allowed a comparison of radiographic findings obtained with CT, DR and AR. Despite superimposition of a 3D structure (bones) on a 2D plane [39], conventional and digital radiography are sensitive in depiction of different bone pathologies. Above all, one result is worth specific mentioning: the conventional radiographs were able to depict excellent bone details, regardless of being manually developed. Nevertheless, minor lesions (numerous in rhinoceros 4) or even more extensive lesions surrounded by multiple bones could not be depicted. These findings are in concordance with previous published data in horses [40], [41], [62], [63], reinforcing the conclusion that CT is very useful for diagnosis of subtle bone lesions when radiography remains inconclusive [64], yet in rhinoceroses, being applied only *post mortem*.

## Conclusions

Far from being a wild dream, imaging in wild animals has been advancing in fits comprehending that improved knowledge of radiologic diagnosis is important for animals' welfare. Our study makes known by what means synchronized computed tomography- digital radiography provides manifold diagnostic tools, a novel perspective and major advances in wildlife's diagnostic imaging.

Putting all together, it is highly recommended that foot radiographic examination became a standard diagnostic technique and, ideally, also a periodic monitoring tool in captive wild animals. Radiographic investigations counted as highly diagnostic and non-invasive procedures should be relied upon when developing the most appropriate wildlife management and conservation strategies.

## Supporting Information

Figure S1 **Additional radiographic exposure chart for front and hind feet in both Southern white and Indian rhinoceroses.** On the horizontal axis are the eight radiographic views and the vertical axis shows the exposure values of: milliampere (mA), kilovolt peak (kVp) and time (s) for each projection at a constant source-to-film or focus-to-film distance (FFD) of 100 cm. Standard radiographic views were: DP [dorso-palmar (plantar)], DM-PL [dorsomedial-palmaro (plantaro) lateral], ML [medio-lateral], PM-DL [palmaro (plantaro) medial-dorsolateral], PD [palmaro (plantaro)-dorsal], PL-DM [palmaro (plantaro) lateral-dorsomedial]; LM [latero-medial], DL-PM [dorsolateral-palmaro (plantaro) medial].
(TIF)

Figure S2 **Dorso-palmar (DP) orthogonal view performed at a projection angle of 0° from the dorsal mid-plane (arrow).** Positioning technique is demonstrated on three-dimensional computed tomographic (3D CT) images of Indian rhinoceros 3 right front foot (right side image) and schematically represented using a cross-sectional CT image (left side image). Semi-transparent 3D CT imaging protocol was employed to show both foot's exterior aspect and the underlying bony structures.
(TIF)

Figure S3 **Medio-lateral (ML) 80° view performed at a projection angle of 80° from the dorsal mid-plane (arrow) allows a better visualization of all digits than the traditional ML 90° orthogonal view.** Positioning technique is demonstrated on three-dimensional computed tomographic (3D CT) images of Indian rhinoceros 3 right front foot (right side image) and schematically represented using a cross-sectional CT image (left side image). Semi-transparent 3D CT imaging protocol was employed to show both foot's exterior aspect and the underlying bony structures.
(TIF)

Figure S4 **Palmaromedial-dorsolateral (PMDL) 150° oblique view performed at a projection angle of 150° from the dorsal mid-plane (arrow) allows a better visualization of all digits than the traditional PMDL 135° oblique view.** Positioning technique is demonstrated on three-dimensional computed tomographic (3D CT) images of Indian rhinoceros 3 right front foot (right side image) and schematically represented using a cross-sectional CT image (left side image). Semi-transparent 3D CT imaging protocol was employed to show both foot's exterior aspect and the underlying bony structures.
(TIF)

Figure S5 **Palmaro-dorsal (PD) 180° orthogonal view performed at a projection angle of 180° from the dorsal mid-plane (arrow) is identical with the traditional DMPL 180° orthogonal view.** Positioning technique is demonstrated on three-dimensional computed tomographic (3D CT) images of Indian rhinoceros 3 right front foot (right side image) and schematically represented using a cross-sectional CT image (left side image). Semi-transparent 3D CT imaging protocol was employed to show both foot's exterior aspect and the underlying bony structures.
(TIF)

Figure S6 **Palmarolateral-dorsomedial (PLDM) 200° oblique view performed at a projection angle of 200° from the dorsal mid-plane (arrow) allows a better visualization of all digits than the traditional PLDM 225° oblique view.** Positioning technique is demonstrated on three-dimensional computed tomographic (3D CT) images of Indian rhinoceros 3 right front foot (right side image) and schematically represented using a cross-sectional CT image (left side image). Semi-transparent 3D CT imaging protocol was employed to show both foot's exterior aspect and the underlying bony structures.
(TIF)

Figure S7 **Latero-medial (LM) 270° orthogonal view performed at a projection angle of 270° from the dorsal mid-plane (arrow) is identical with the traditional LM 270° orthogonal view.** Positioning technique is demonstrated on three-dimensional computed tomographic (3D CT) images of Indian rhinoceros 3 right front foot (right side image) and schematically represented using a cross-sectional CT image (left side image). Semi-transparent 3D CT imaging protocol was employed to show both foot's exterior aspect and the underlying bony structures.
(TIF)

Figure S8 **Dorsolateral-palmaromedial (DLPM) 330° oblique view performed at a projection angle of 330° from the dorsal mid-plane (arrow) allows a better visualization of all digits than the traditional DLPM 315° oblique view.** Positioning technique is demonstrated on three-dimensional computed tomographic (3D CT) images of Indian rhinoceros 3 right front foot (right side image) and schematically represented using a cross-sectional CT image (left side image). Semi-transparent 3D CT imaging protocol was employed to show both foot's exterior aspect and the underlying bony structures.
(TIF)

Figure S9 **Osteolysis and bone rarefaction (circle) in rhinoceros 2 left front foot on the distal metacarpal bone and first phalanx of the second (medial) digit.** These pathologies are visualized by synchronized computed tomography (A) and digital radiography (B).
(TIF)

Figure S10 **Proliferative new bone formation and bone remodeling anatomy (circle) depicted in Southern white rhinoceros 2 right front foot-palmar aspect (P) by (A) computed tomography (CT) and (B) synchronized digital radiography (Synch DR).**
(TIF)

Figure S11 **Intra-articular bony fragment showed in (A) computed tomography (CT) and (B) synchronized digital radiography (Synch DR) of Indian rhinoceros 1 left front foot.** This bony fragment (circle) has smooth margins and is situated on the lateral aspect of the central digit between the metacarpus and the first phalanx.
(TIF)

Figure S12 **Bone pathology (circle) demonstrated in left tarsal joint in rhinoceros 1 by means of (A) synchronized digital radiography (Synch DR) and (B) computed tomography (CT).** Left central tarsal bone (CTB) fractures are concealed by new bone production and, therefore, undetectable on three-dimensional CT images, but visible on Synch DR images. At the level of these fractures, CTB distalo-medial aspect reveals a mixed pattern of trabecular focal bone loss (osteolysis) and cortical osteogenesis represented by massive, unstructured new bone production and remodeling, with a beak-like formation oriented plantaro-medially, hook-shaped (circle). Additionally, the articular surface between CTB and first tarsal bone (TI) is highly irregular, characterized by decreased joint space width, articular bone proliferation that bridges the contiguous bones (ankylosis), erosion and lysis of the articular cartilage and underlying bone (asterisk).
(TIF)

Figure S13 **Periosteal proliferation demonstrated in rhinoceros 1 left hind foot, on the lateral aspect of the second metatarsal bone (circle) by (A) computed tomography (CT) and (B) synchronized digital radiography (Synch DR).**
(TIF)
