# Supplementary figures and images for: Rhinoceros Feet Step Out of a Rule-of-Thumb: A Wildlife Imaging Pioneering Approach of Synchronized Computed Tomography-Digital Radiography
Source: PLoS One. 2014 Jun 25;9(6):e100415. doi: 10.1371/journal.pone.0100415 (PMC4070926; doi:10.1371/journal.pone.0100415)

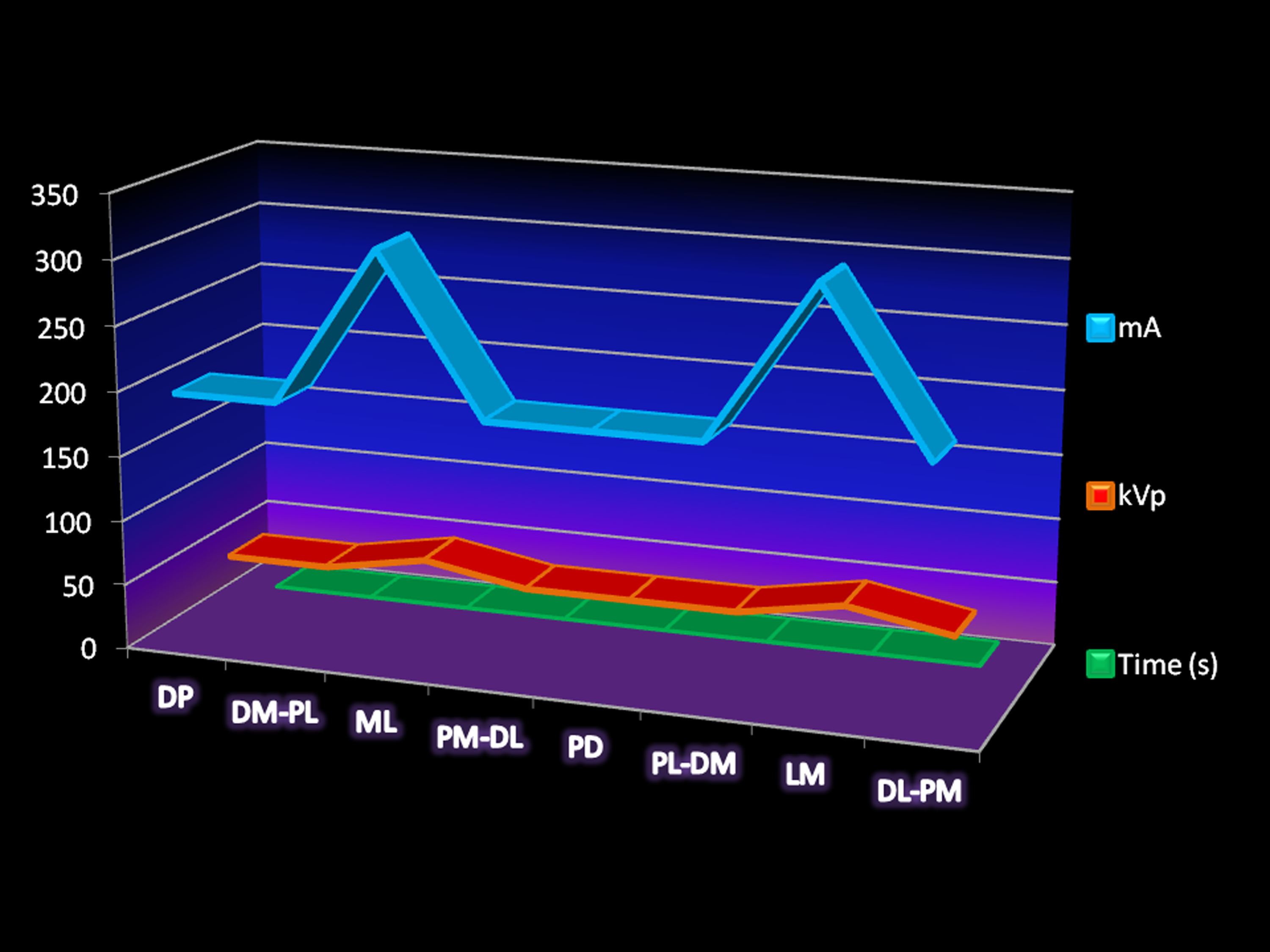

Supplement: Figure S1 — Additional radiographic exposure chart for front and hind feet in both Southern white and Indian rhinoceroses. On the horizontal axis are the eight radiographic views and the vertical axis shows the exposure values of: milliampere (mA), kilovolt peak (kVp) and time (s) for each projection at a constant source-to-film or focus-to-film distance (FFD) of 100 cm. Standard radiographic views were: DP [dorso-palmar (plantar)], DM-PL [dorsomedial-palmaro (plantaro) lateral], ML [medio-lateral], PM-DL [palmaro (plantaro) medial-dorsolateral], PD [palmaro (plantaro)-dorsal], PL-DM [palmaro (plantaro) lateral-dorsomedial]; LM [latero-medial], DL-PM [dorsolateral-palmaro (plantaro) medial]. (TIF) [file pone.0100415.s001.tif]

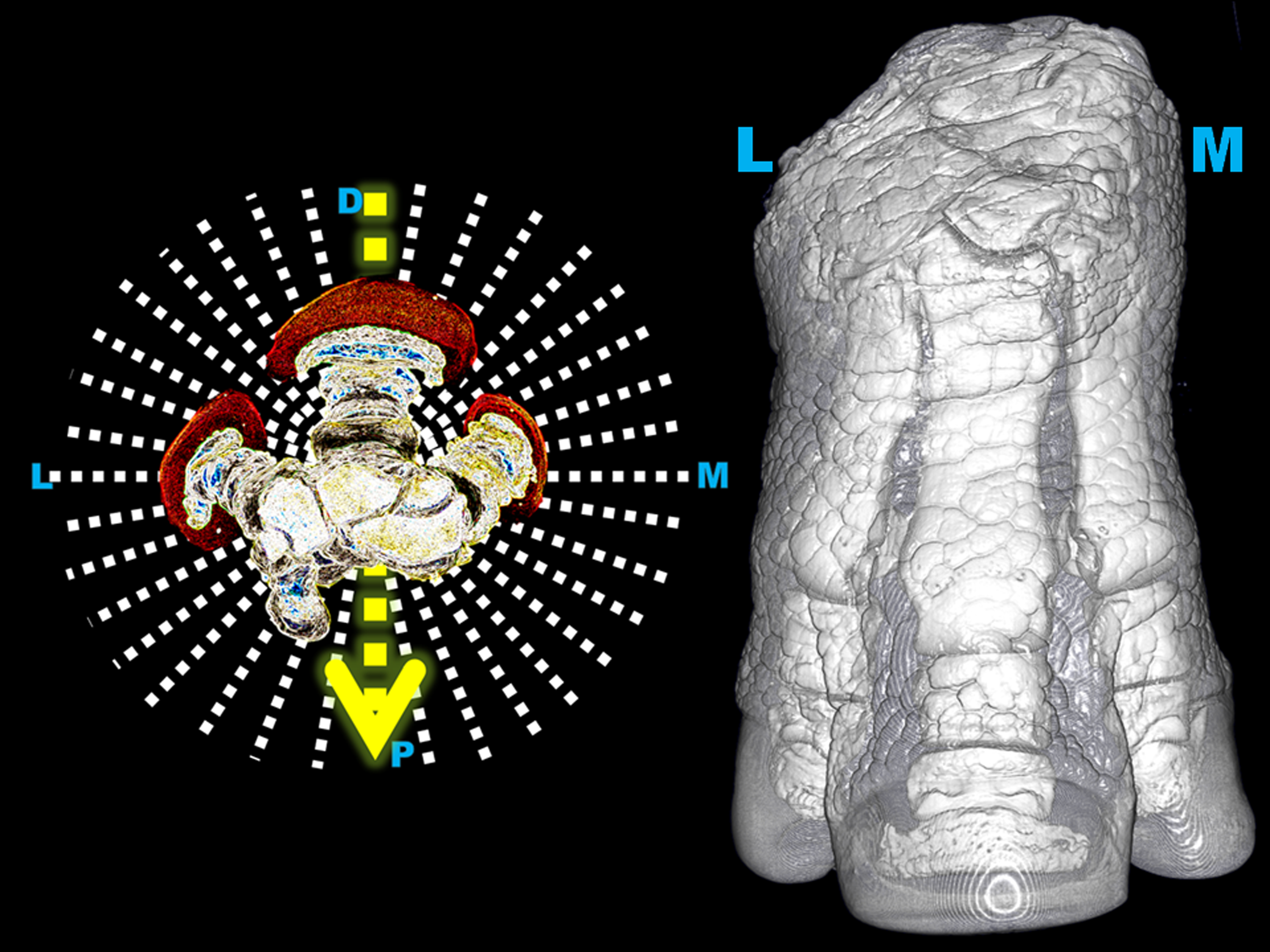

Supplement: Figure S2 — Dorso-palmar (DP) orthogonal view performed at a projection angle of 0° from the dorsal mid-plane (arrow). Positioning technique is demonstrated on three-dimensional computed tomographic (3D CT) images of Indian rhinoceros 3 right front foot (right side image) and schematically represented using a cross-sectional CT image (left side image). Semi-transparent 3D CT imaging protocol was employed to show both foot's exterior aspect and the underlying bony structures. (TIF) [file pone.0100415.s002.tif]

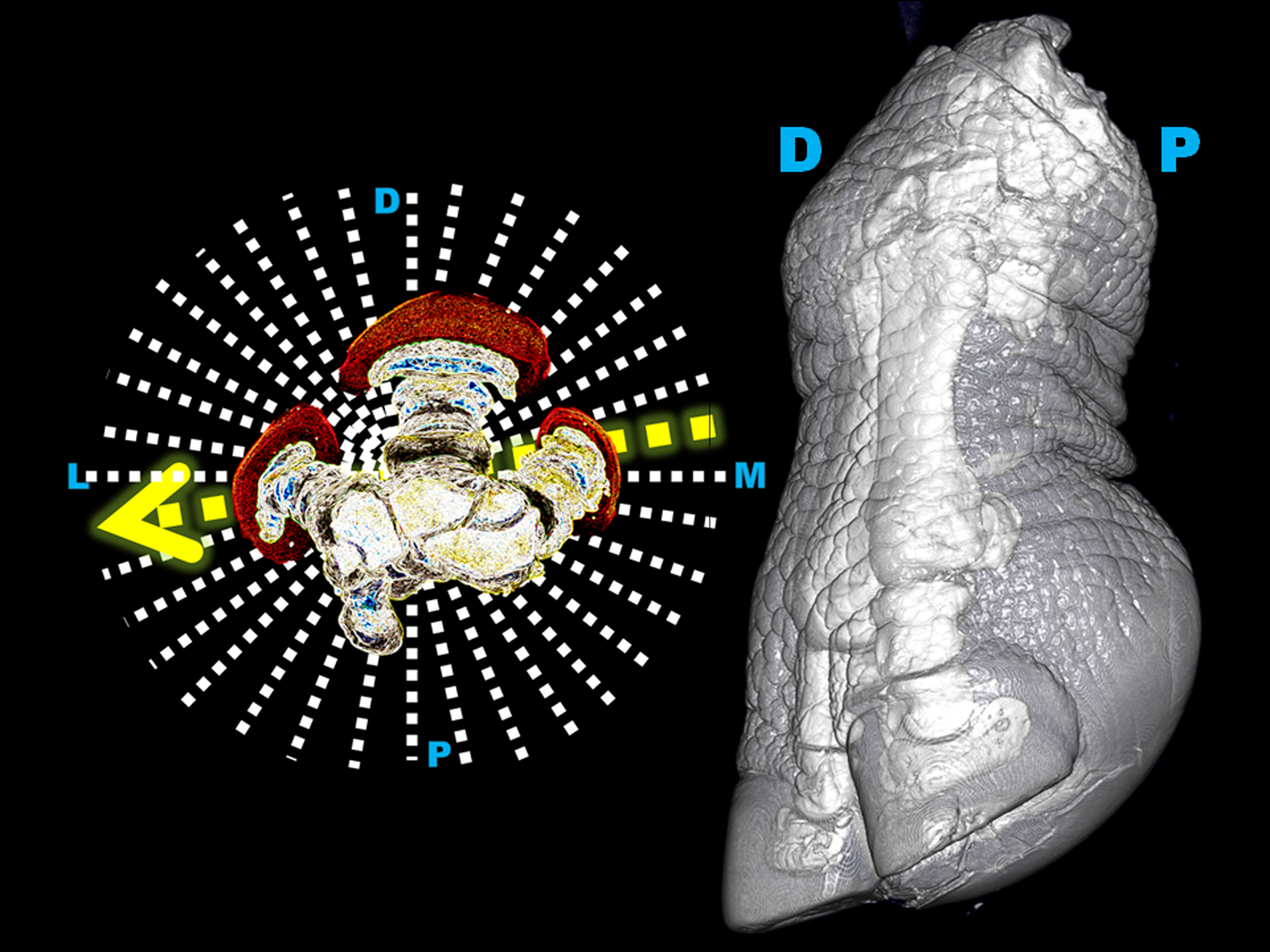

Supplement: Figure S3 — Medio-lateral (ML) 80° view performed at a projection angle of 80° from the dorsal mid-plane (arrow) allows a better visualization of all digits than the traditional ML 90° orthogonal view. Positioning technique is demonstrated on three-dimensional computed tomographic (3D CT) images of Indian rhinoceros 3 right front foot (right side image) and schematically represented using a cross-sectional CT image (left side image). Semi-transparent 3D CT imaging protocol was employed to show both foot's exterior aspect and the underlying bony structures. (TIF) [file pone.0100415.s003.tif]

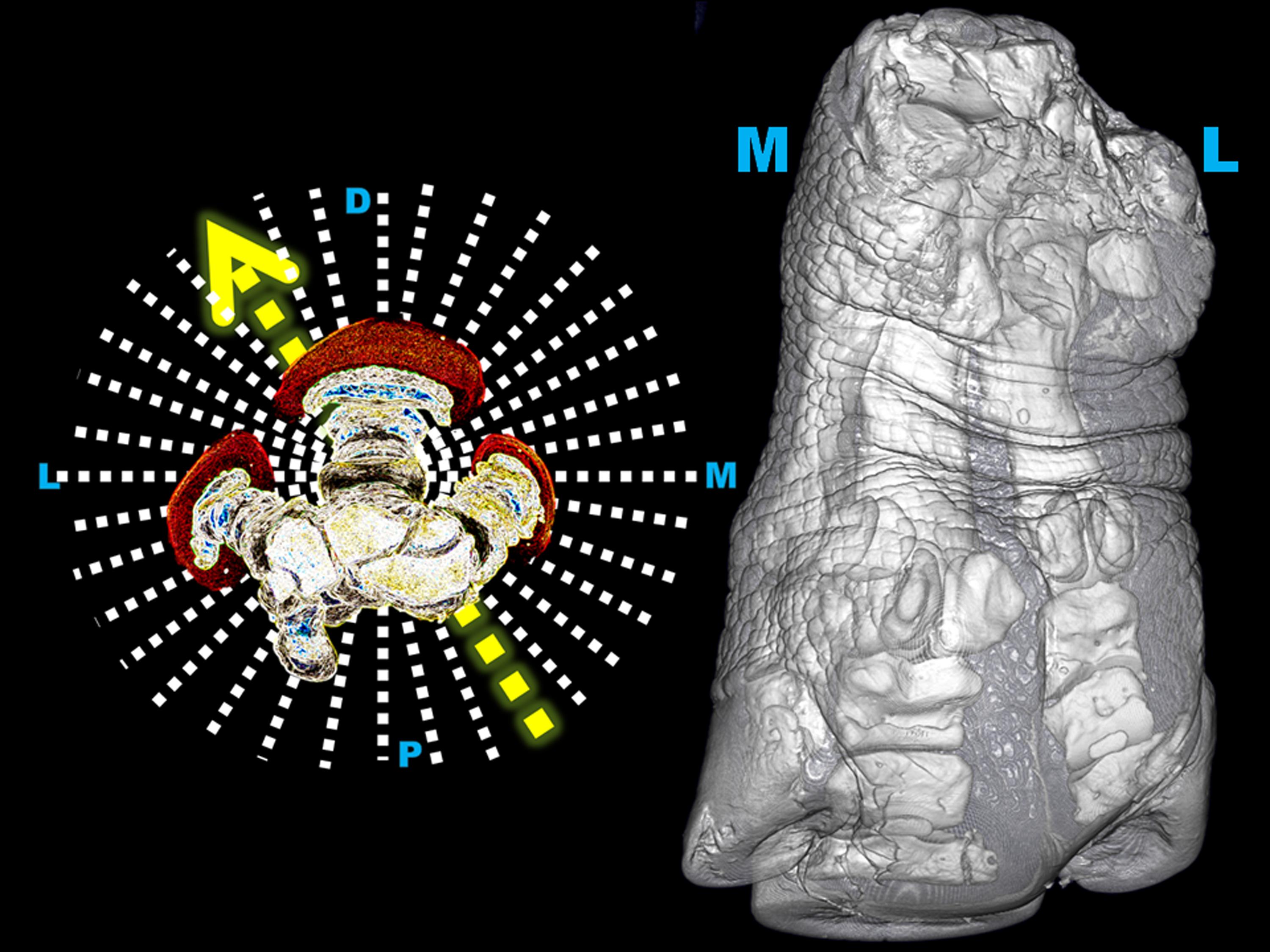

Supplement: Figure S4 — Palmaromedial-dorsolateral (PMDL) 150° oblique view performed at a projection angle of 150° from the dorsal mid-plane (arrow) allows a better visualization of all digits than the traditional PMDL 135° oblique view. Positioning technique is demonstrated on three-dimensional computed tomographic (3D CT) images of Indian rhinoceros 3 right front foot (right side image) and schematically represented using a cross-sectional CT image (left side image). Semi-transparent 3D CT imaging protocol was employed to show both foot's exterior aspect and the underlying bony structures. (TIF) [file pone.0100415.s004.tif]

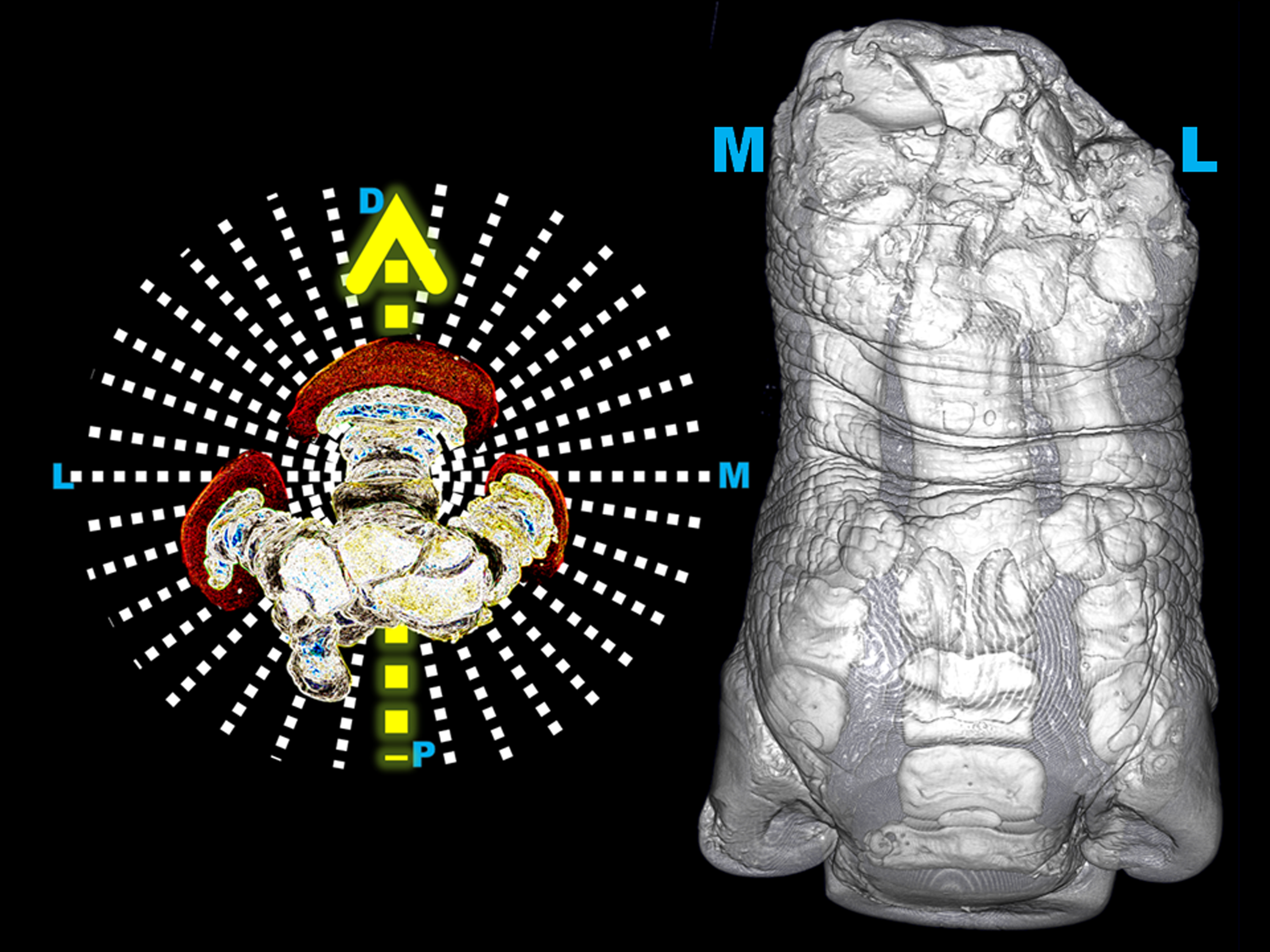

Supplement: Figure S5 — Palmaro-dorsal (PD) 180° orthogonal view performed at a projection angle of 180° from the dorsal mid-plane (arrow) is identical with the traditional DMPL 180° orthogonal view. Positioning technique is demonstrated on three-dimensional computed tomographic (3D CT) images of Indian rhinoceros 3 right front foot (right side image) and schematically represented using a cross-sectional CT image (left side image). Semi-transparent 3D CT imaging protocol was employed to show both foot's exterior aspect and the underlying bony structures. (TIF) [file pone.0100415.s005.tif]

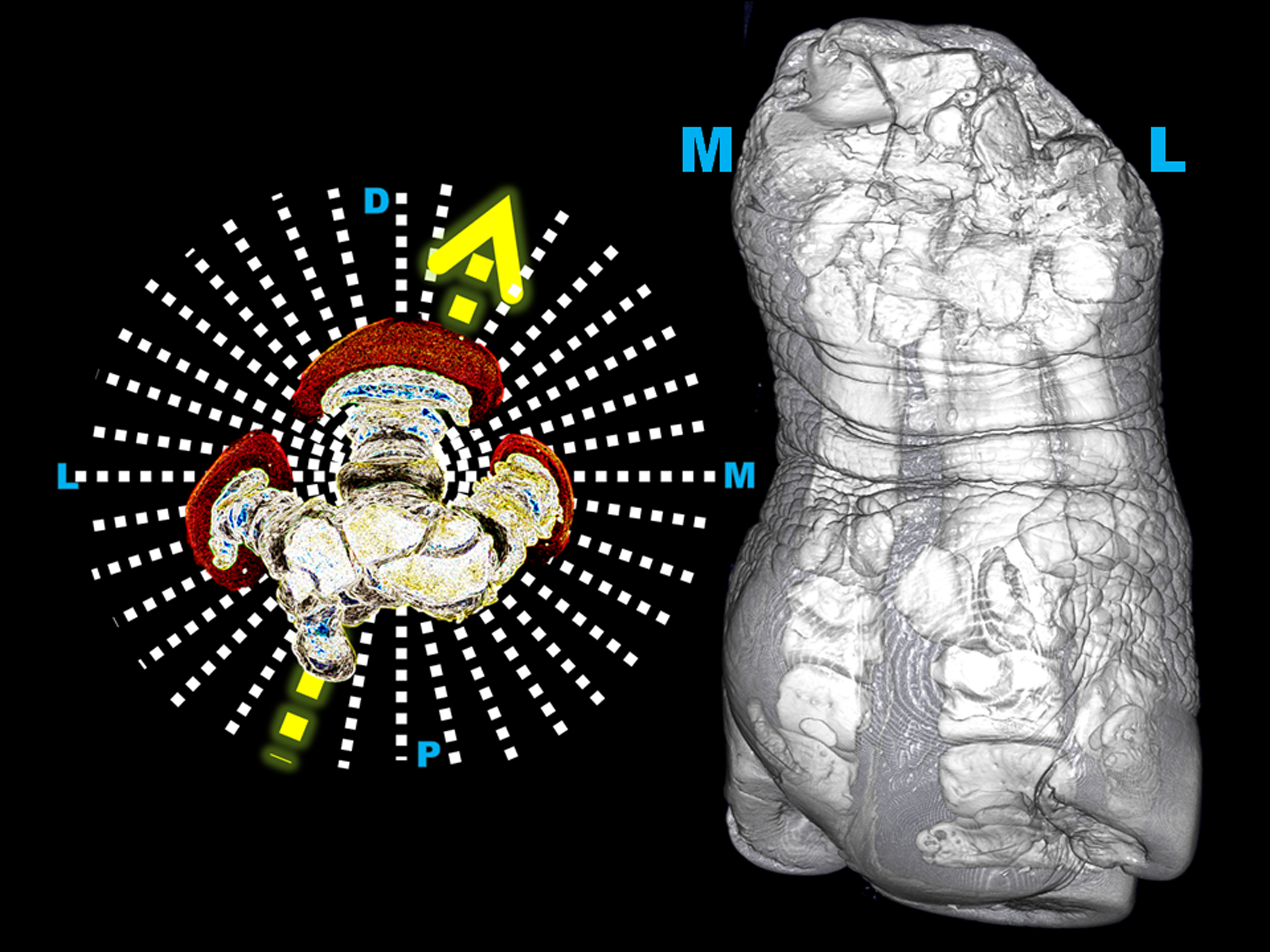

Supplement: Figure S6 — Palmarolateral-dorsomedial (PLDM) 200° oblique view performed at a projection angle of 200° from the dorsal mid-plane (arrow) allows a better visualization of all digits than the traditional PLDM 225° oblique view. Positioning technique is demonstrated on three-dimensional computed tomographic (3D CT) images of Indian rhinoceros 3 right front foot (right side image) and schematically represented using a cross-sectional CT image (left side image). Semi-transparent 3D CT imaging protocol was employed to show both foot's exterior aspect and the underlying bony structures. (TIF) [file pone.0100415.s006.tif]

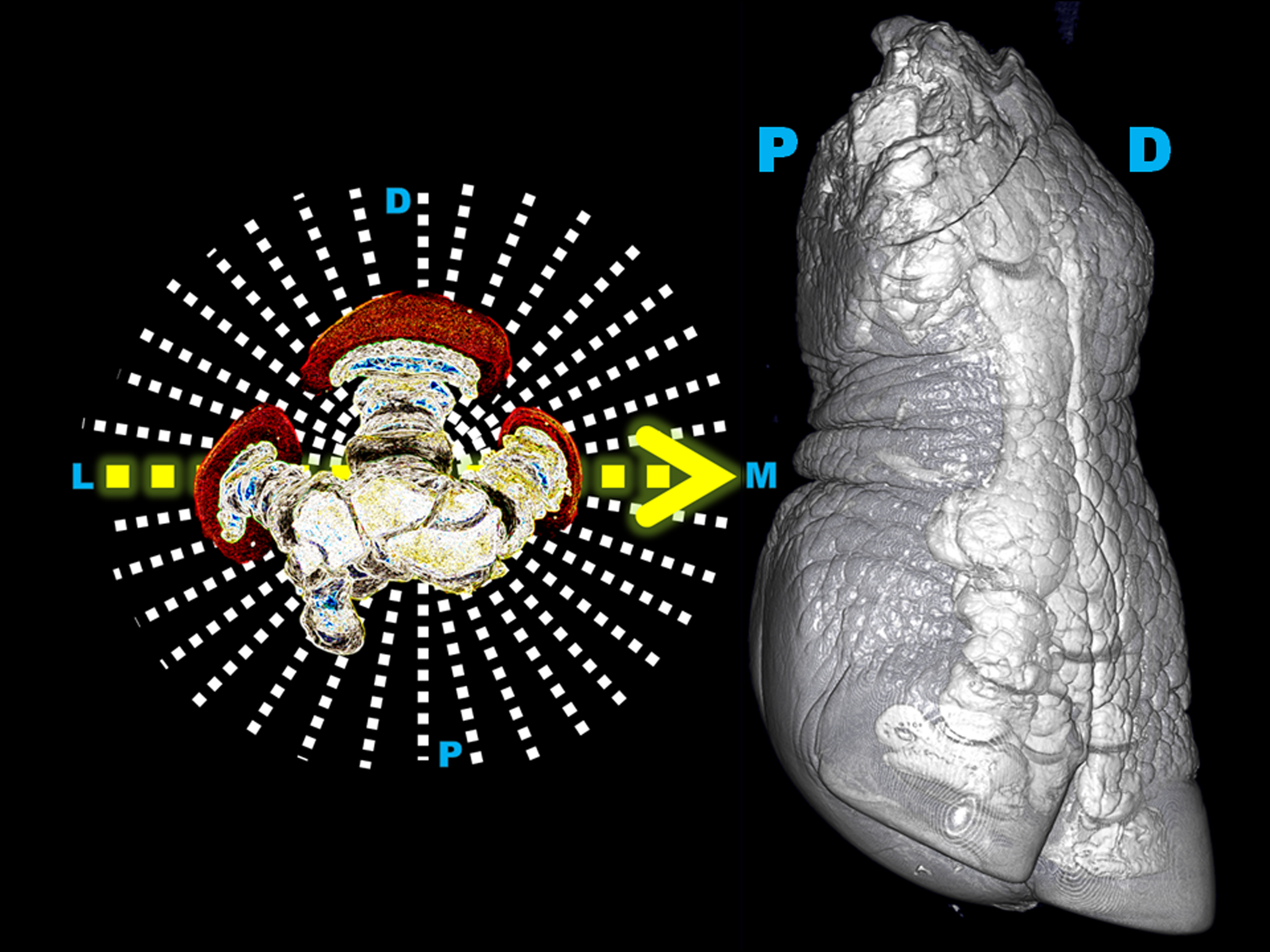

Supplement: Figure S7 — Latero-medial (LM) 270° orthogonal view performed at a projection angle of 270° from the dorsal mid-plane (arrow) is identical with the traditional LM 270° orthogonal view. Positioning technique is demonstrated on three-dimensional computed tomographic (3D CT) images of Indian rhinoceros 3 right front foot (right side image) and schematically represented using a cross-sectional CT image (left side image). Semi-transparent 3D CT imaging protocol was employed to show both foot's exterior aspect and the underlying bony structures. (TIF) [file pone.0100415.s007.tif]

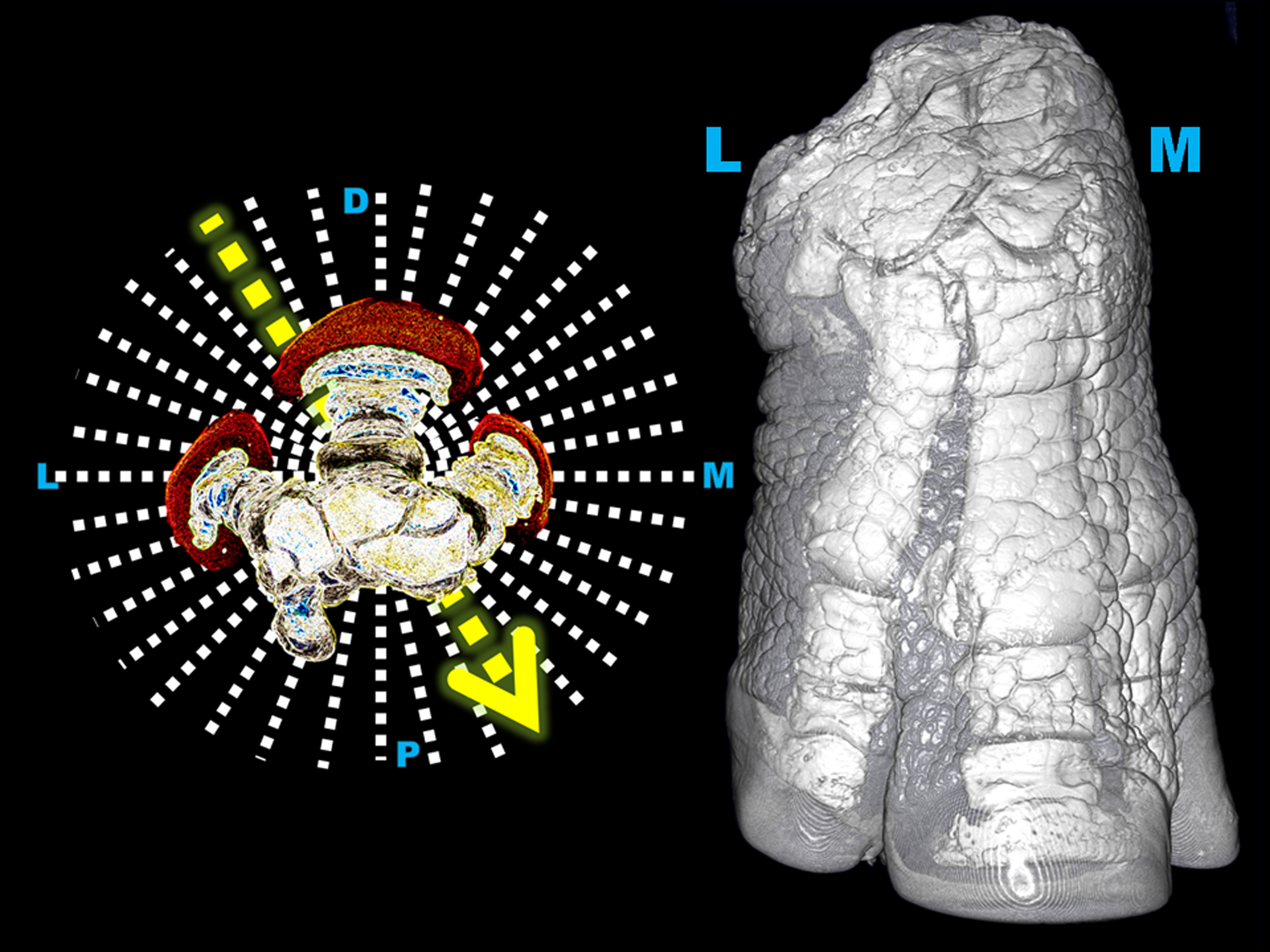

Supplement: Figure S8 — Dorsolateral-palmaromedial (DLPM) 330° oblique view performed at a projection angle of 330° from the dorsal mid-plane (arrow) allows a better visualization of all digits than the traditional DLPM 315° oblique view. Positioning technique is demonstrated on three-dimensional computed tomographic (3D CT) images of Indian rhinoceros 3 right front foot (right side image) and schematically represented using a cross-sectional CT image (left side image). Semi-transparent 3D CT imaging protocol was employed to show both foot's exterior aspect and the underlying bony structures. (TIF) [file pone.0100415.s008.tif]

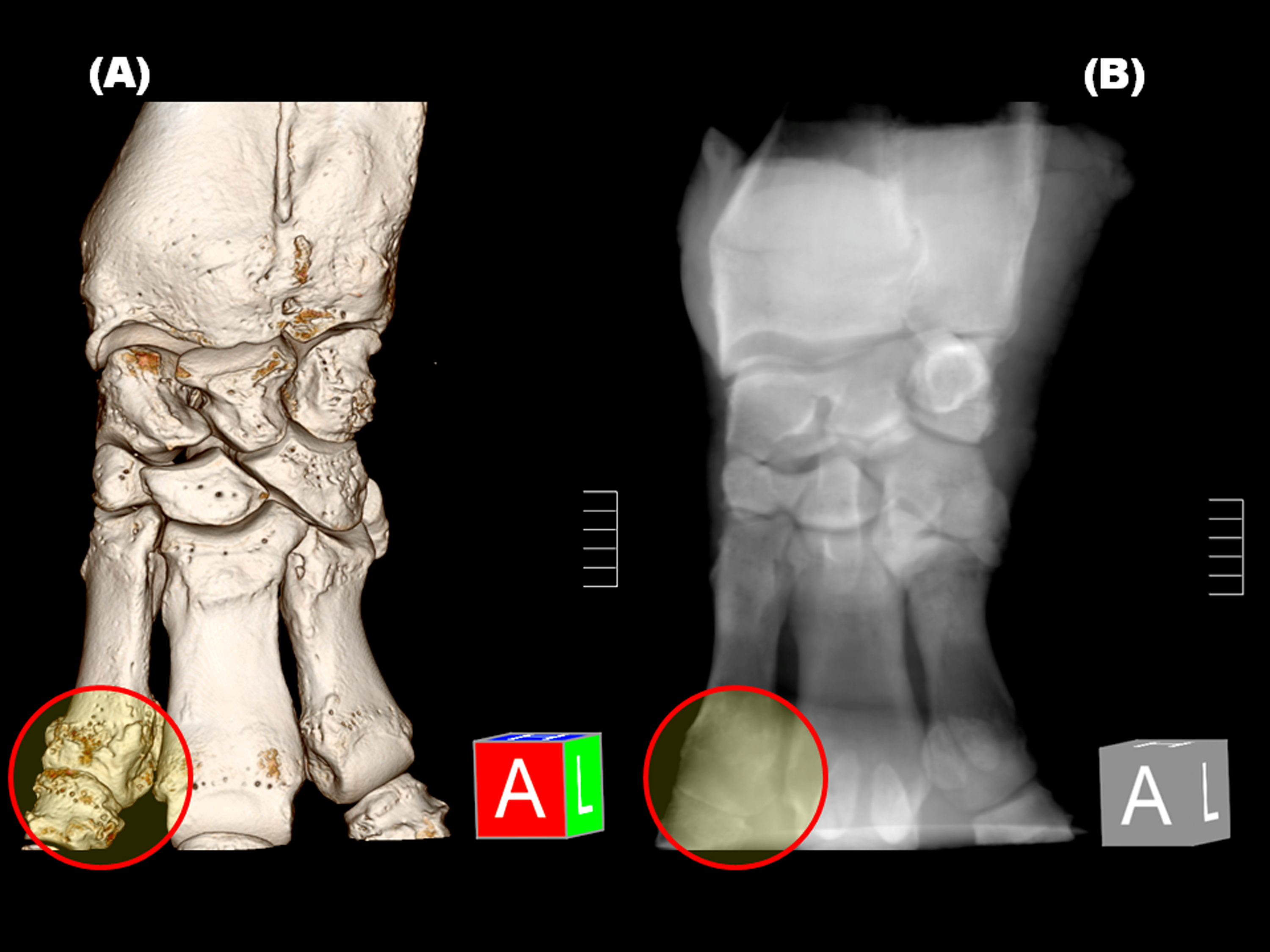

Supplement: Figure S9 — Osteolysis and bone rarefaction (circle) in rhinoceros 2 left front foot on the distal metacarpal bone and first phalanx of the second (medial) digit. These pathologies are visualized by synchronized computed tomography (A) and digital radiography (B). (TIF) [file pone.0100415.s009.tif]

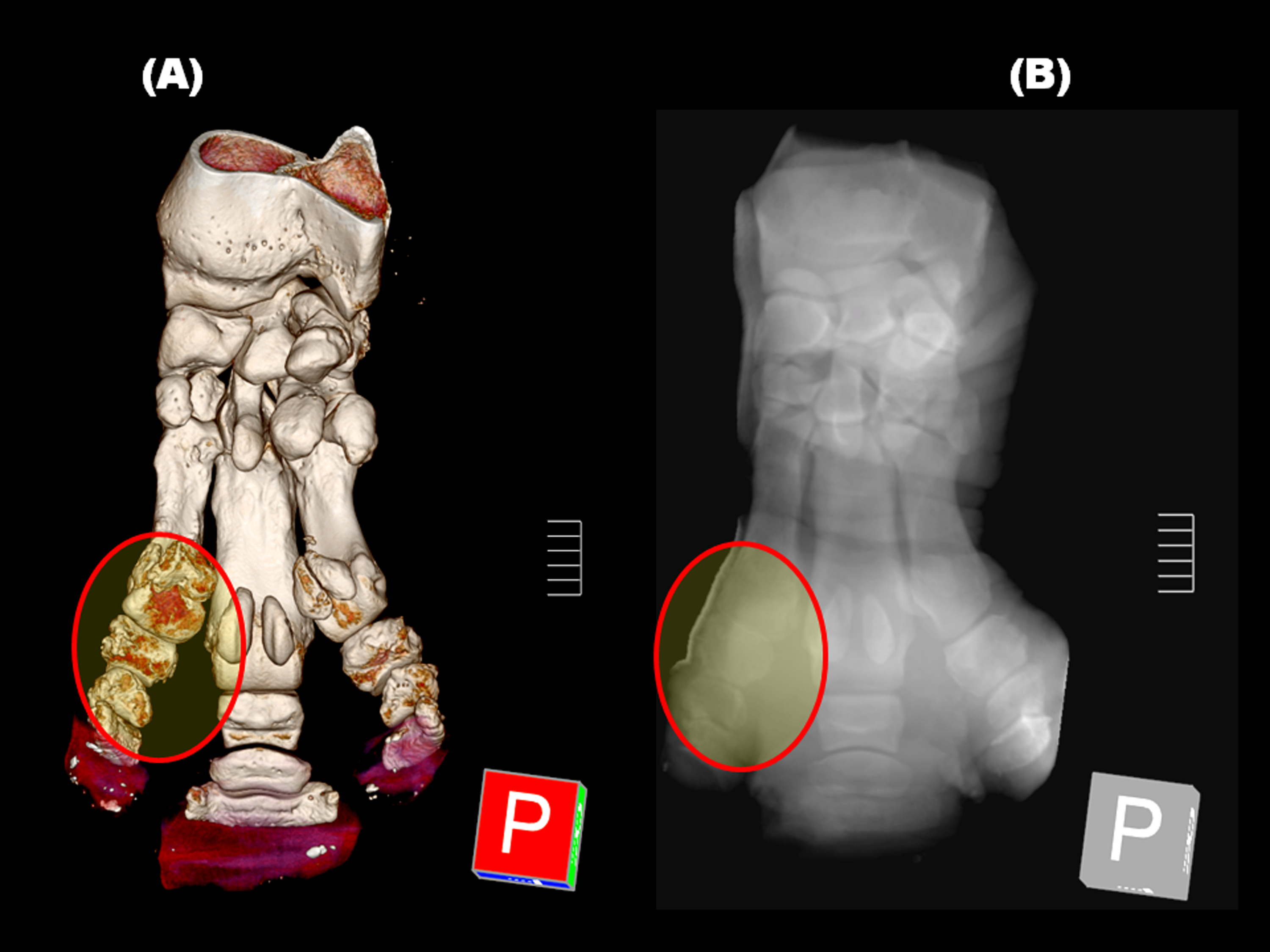

Supplement: Figure S10 — Proliferative new bone formation and bone remodeling anatomy (circle) depicted in Southern white rhinoceros 2 right front foot-palmar aspect (P) by (A) computed tomography (CT) and (B) synchronized digital radiography (Synch DR). (TIF) [file pone.0100415.s010.tif]

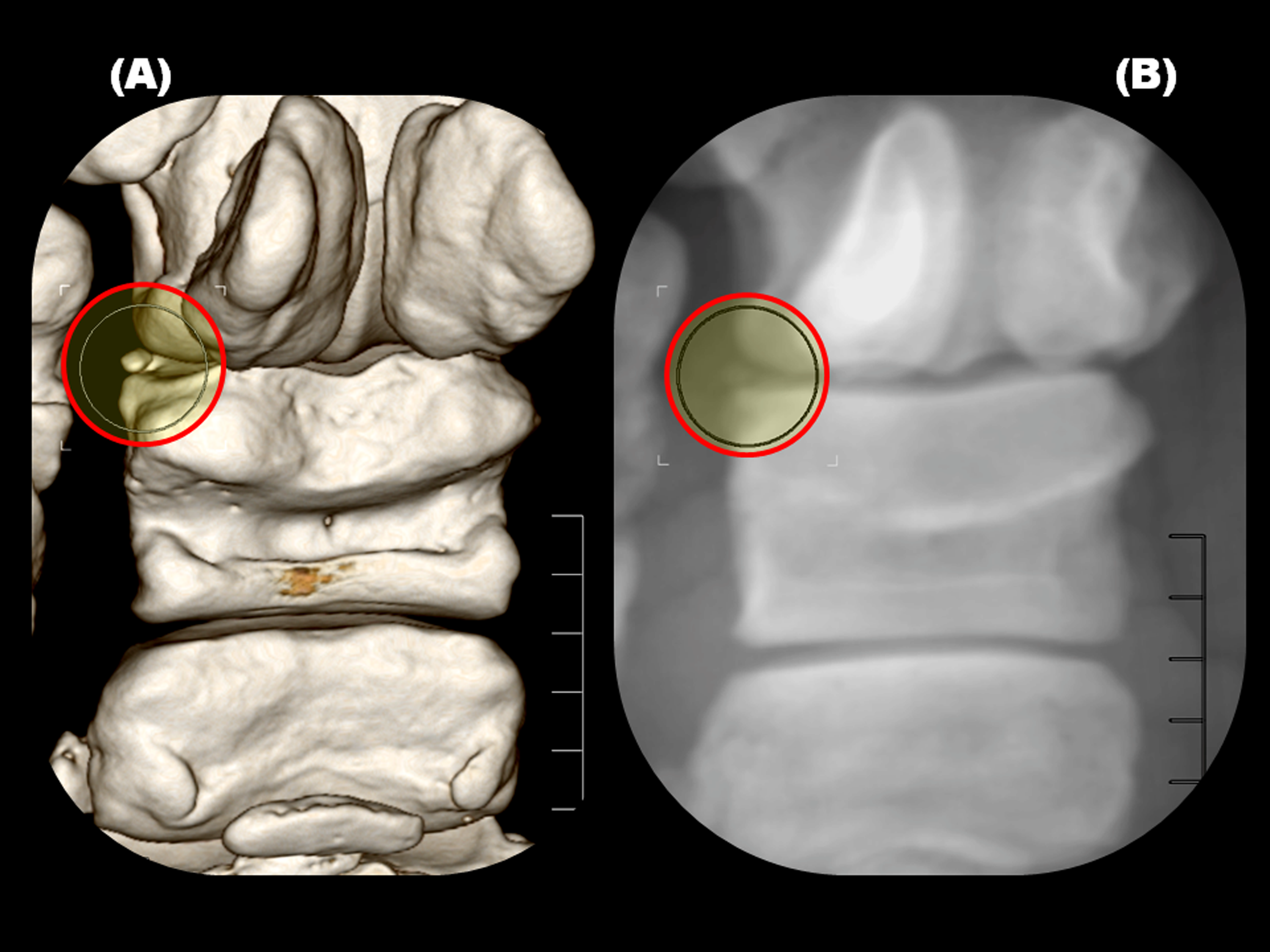

Supplement: Figure S11 — Intra-articular bony fragment showed in (A) computed tomography (CT) and (B) synchronized digital radiography (Synch DR) of Indian rhinoceros 1 left front foot. This bony fragment (circle) has smooth margins and is situated on the lateral aspect of the central digit between the metacarpus and the first phalanx. (TIF) [file pone.0100415.s011.tif]

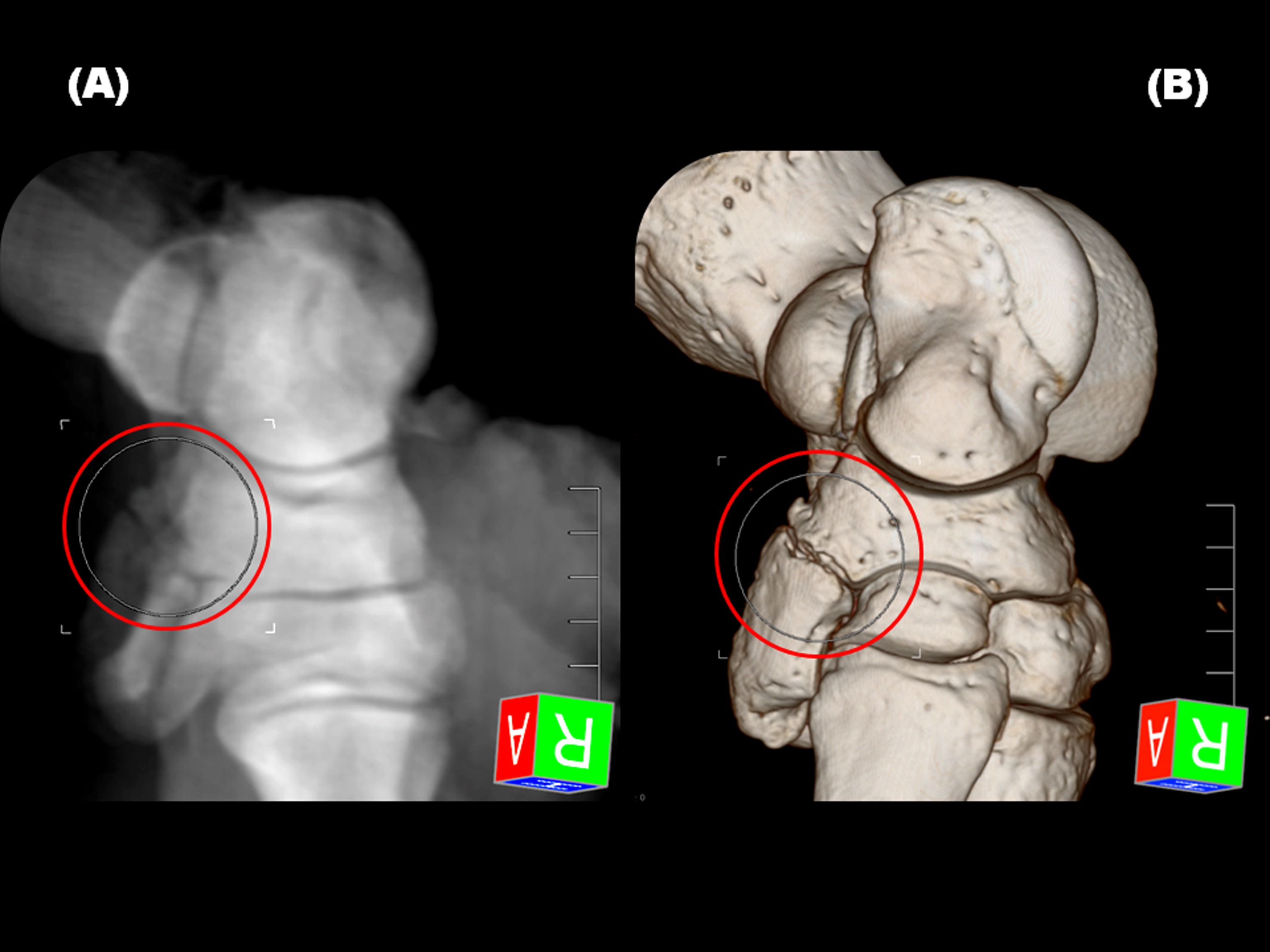

Supplement: Figure S12 — Bone pathology (circle) demonstrated in left tarsal joint in rhinoceros 1 by means of (A) synchronized digital radiography (Synch DR) and (B) computed tomography (CT). Left central tarsal bone (CTB) fractures are concealed by new bone production and, therefore, undetectable on three-dimensional CT images, but visible on Synch DR images. At the level of these fractures, CTB distalo-medial aspect reveals a mixed pattern of trabecular focal bone loss (osteolysis) and cortical osteogenesis represented by massive, unstructured new bone production and remodeling, with a beak-like formation oriented plantaro-medially, hook-shaped (circle). Additionally, the articular surface between CTB and first tarsal bone (TI) is highly irregular, characterized by decreased joint space width, articular bone proliferation that bridges the contiguous bones (ankylosis), erosion and lysis of the articular cartilage and underlying bone (asterisk). (TIF) [file pone.0100415.s012.tif]

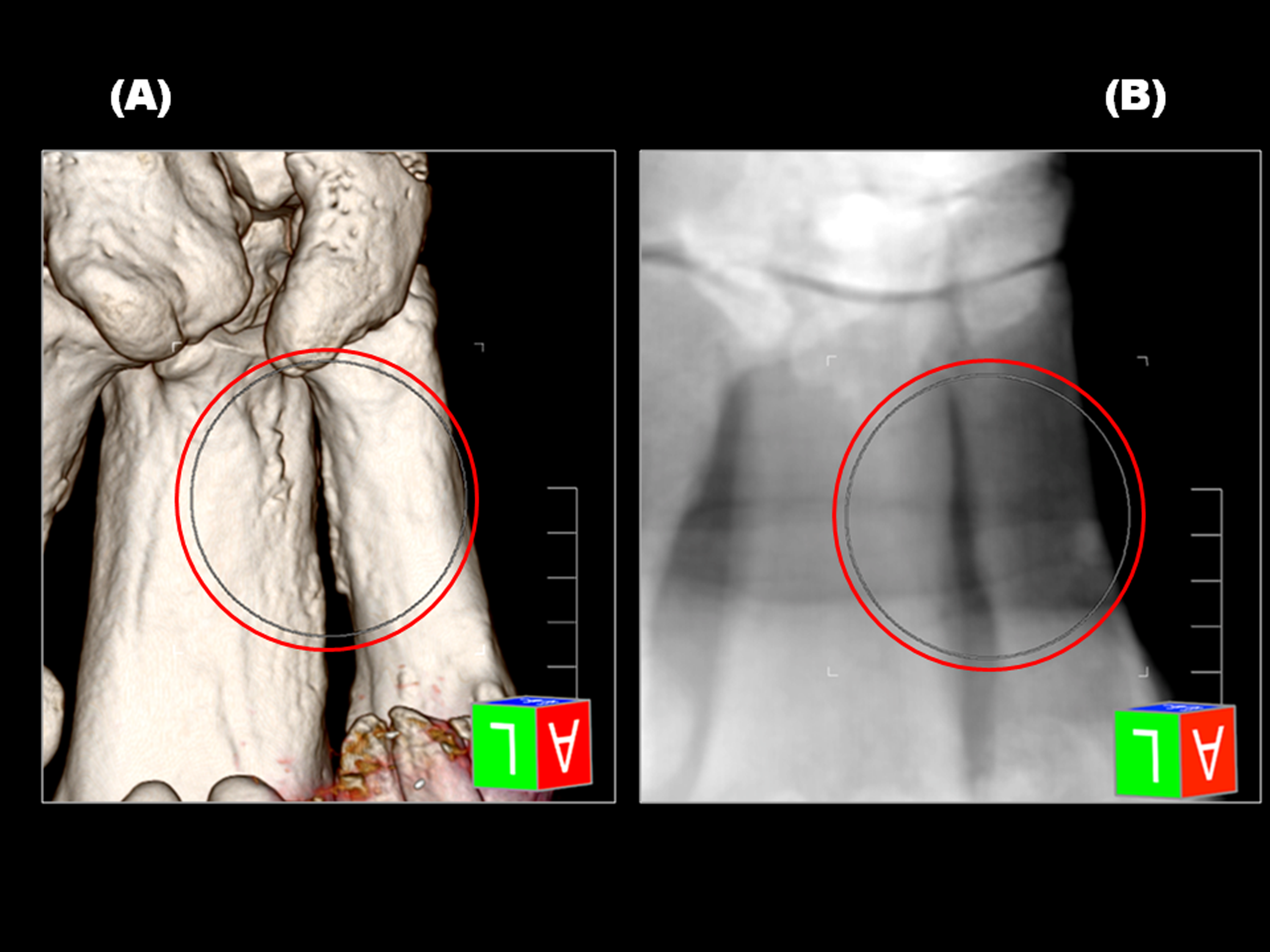

Supplement: Figure S13 — Periosteal proliferation demonstrated in rhinoceros 1 left hind foot, on the lateral aspect of the second metatarsal bone (circle) by (A) computed tomography (CT) and (B) synchronized digital radiography (Synch DR). (TIF) [file pone.0100415.s013.tif]
